# Supplementary material for: Green Synthesis of Transition-Metal Nanoparticles and Their Oxides: A Review
Source: Materials (Basel). 2021 May 21;14(11):2700. doi: 10.3390/ma14112700 (PMC8196554; doi:10.3390/ma14112700)
Supplement: Supplementary file 1 [file materials-14-02700-s001.zip › materials-1130148-supplementary.pdf]

materials

Supplementary Material

Green Synthesis of Transition-Metal Nanoparticles and Their Oxides: A Review

Sean Drummer, Tafirenyika Madzimbamuto and Mahabubur Chowdhury *

Department of Chemical Engineering, Cape Peninsula University of Technology, Symphony way, Bellville 7535, South Africa; sndrummer512@gmail.com (S.D.); MadzimbamutoT@cput.ac.za (T.M.)
* Correspondence: chowdhurym@cput.ac.za

Publisher's Note: MDPI stays neutral with regard to jurisdictional claims in published maps and institutional affiliations.

Copyright: © 2021 by the authors. Licensee MDPI, Basel, Switzerland. This article is an open access article distributed under the terms and conditions of the Creative Commons Attribution (CC BY) license (http://creativecommons.org/licenses/by/4.0/).

Table S1. The Subclasses of Terpenoids.

Class	Example Name	Structure	No. of Isoprene Units	General Formula
Hemiterpenoids	Isoprene	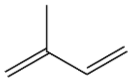	1	C_5H_8
Monoterpenoids	Myrcene	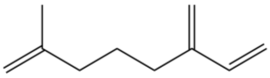	2	$C_{10}H_{16}$
Sesquiterpenoids	α-Himachalene	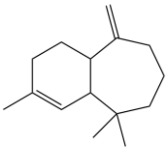	3	$C_{15}H_{24}$
Diterpenoids	Tanshinone	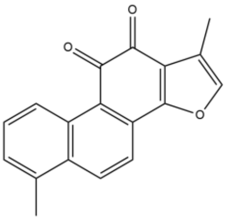	4	$C_{20}H_{32}$
Sesterterpenoids	Ophiobolin A	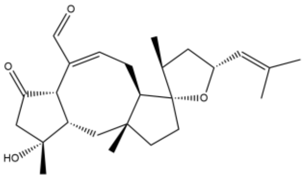	5	$C_{25}H_{40}$
Triterpenoids	Squalene	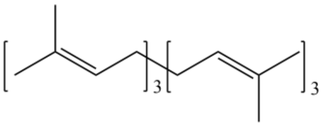	6	$C_{30}H_{48}$
Tetraterpenoids	Cryptoxanthin	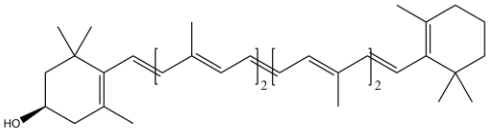	8	$C_{40}H_{64}$
Polyterpenoids	Dolichol	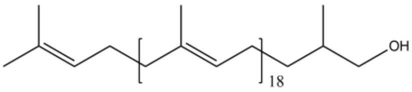	>8	$(C_5H_8)_n$

Table S2. The Subclasses of Phenolics.

Class	Structure	General Formula
	Flavonoids	
Flavones	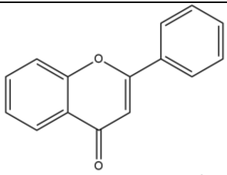	$C_{15}H_{10}O_2$
Flavonols	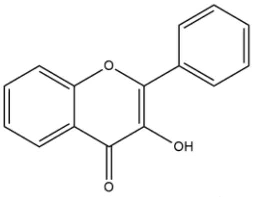	$C_{15}H_{10}O_3$
Flavan-3-ols	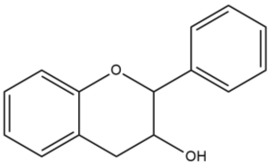	$C_{15}H_{14}O_2$
Flavanones	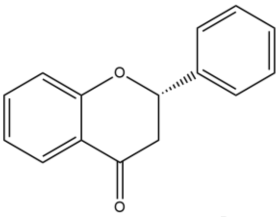	$C_{15}H_{12}O_2$
Isoflavones	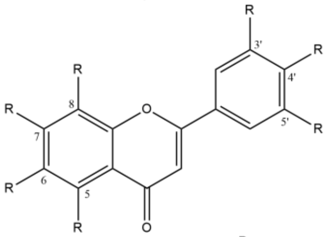	$C_{15}H_{10}O_2$
Anthocyanins	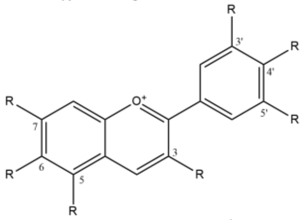	$C_{15}H_{11}O^+$
Chalcones	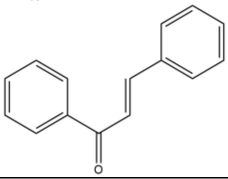	$C_{15}H_{12}O$
	Non-Flavonoids	
Hydroxybenzoic Acids	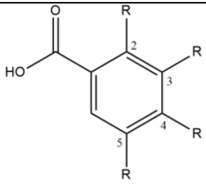	$C_7H_6O_3$

Hydroxycinnamic Acids	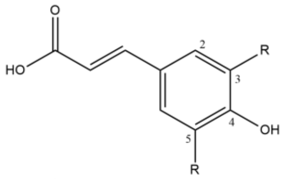	$C_9H_8O_3$
Stilbenes	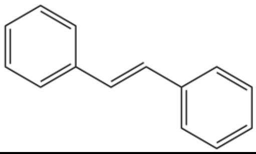	$C_{14}H_{12}$

Table S3. The Subclasses of Alkaloids.

Precursor	Structure	Alkaloid Class
Tryptophan	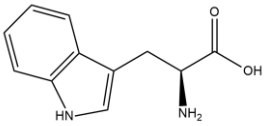	Indole, Terpenoid indole, Quinoline, Pyrroloindole, and Ergot
Phenylalanine	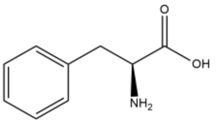	-
Ornithine	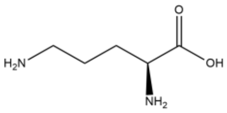	Pyrrolidine, Tropane, and Pyrrolizidine
Tyrosine	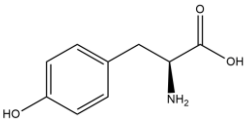	Phenylethylamino- and Isoquinoline
Anthranilic Acid	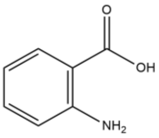	Quinazoline, Quinoline, and Acridine
Histidine	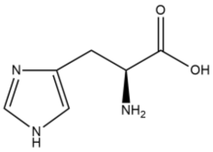	Imidazole
Nicotinic Acid	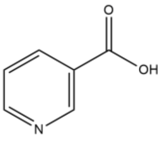	Pyridine

Lysine	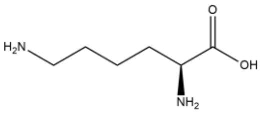	Piperidine, Quinolizidine, Indolizidine
Leucine	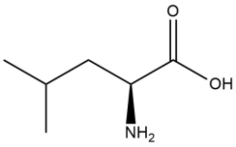	Pyrrole
Purine	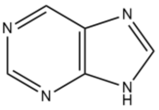	-
Geranylgeranyl-diphosphate	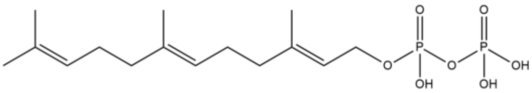	Terpenoidic
Cholesterol	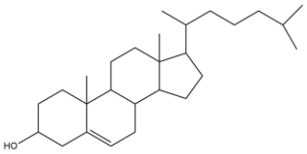	Steroidal

Table S4. The Subclasses of Saponins.

**Class**	**Structure**	**General Formula**
Dammaranes	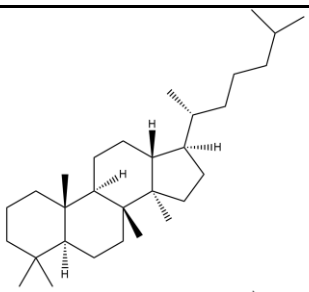	$C_{30}H_{54}$
Tirucallanes	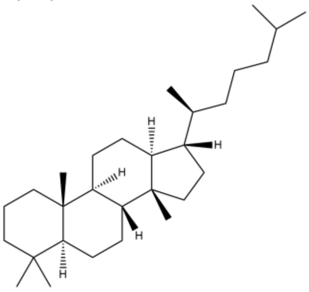	$C_{30}H_{54}$

Lupanes	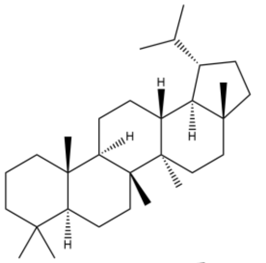	$C_{30}H_{52}$
Hopanes	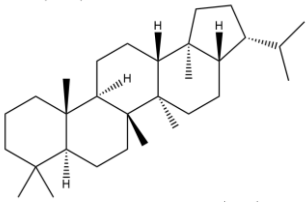	$C_{30}H_{52}$
Oleananes	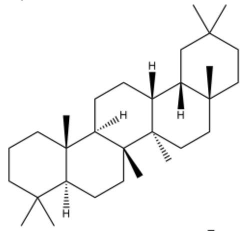	$C_{30}H_{52}$
Taraxastanes	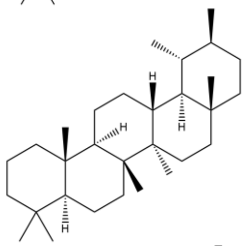	$C_{30}H_{52}$
Ursanes	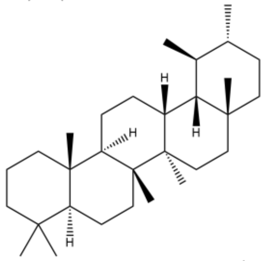	$C_{30}H_{52}$
Cycloartanes	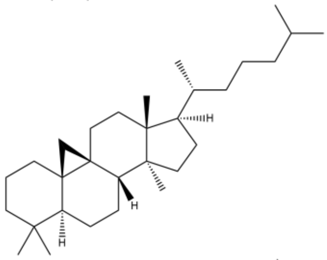	$C_{30}H_{52}$
Lanostanes	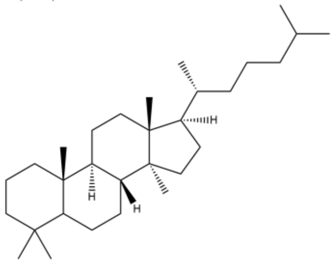	$C_{30}H_{54}$

Cucurbitanes	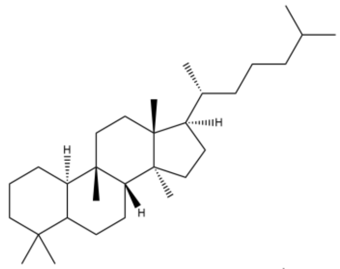	$C_{30}H_{54}$
Steroids	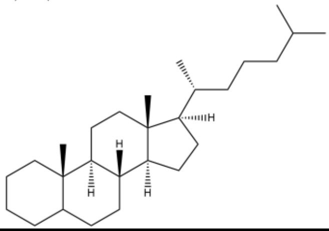	$C_{27}H_{48}$

Table S5. The Subclasses of Carbohydrates.

**Class**	**Name**	**Structure**	**Formula**
Monosaccharides	Glucose	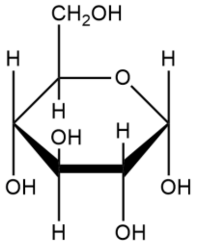	$C_6H_{12}O_6$
Oligosaccharides	Raffinose	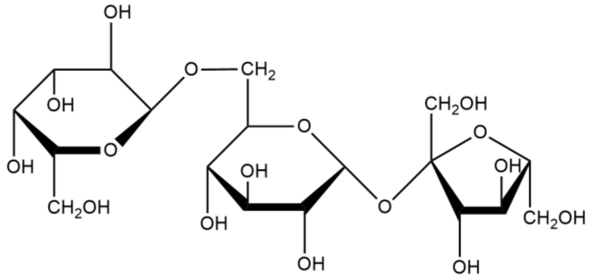	$C_{18}H_{32}O_{16}$
Polysaccharides	Amylose Starch	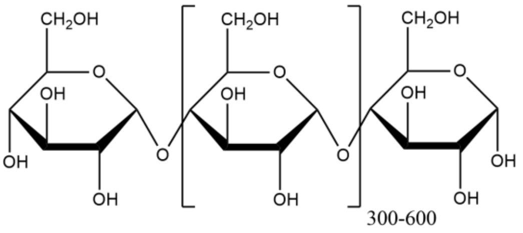	$(C_6H_{10}O_5)_{300\text{-}600}$

Table S6. The Plant-mediated synthesis of Titanium Dioxide Nanoparticles.

Plant Species	Nanoparticle	Particle Size (nm)	Morphology	Characterisation	Application	Reference
Acacia senegal	TiO_2	9	Spherical	XRD, FTIR, Raman, SEM-EDX, TEM, HR-TEM and UV	Organic dye removal	(Saranya et al., 2018a)
Acanthopyllum laxiusculum	TiO_2	20–25	Spherical	XRD, FTIR, EDAX and TEM	-	(Madadi & Lotfabad, 2016)
Ageratina altissima	TiO_2	60–100	Spherical	XRD, FTIR and FSEM	Photocatalytic activity	(Ganesan et al., 2016)
Allium cepa	TiO_2	89–96	-	AFM, SEM, TEM, XRD, UV and FTIR	Antimicrobial activity	(Abdul jalill, 2018)
Aloe vera	TiO_2	30	Irregular	SEM, TEM, XRD, UV and FTIR	-	(Khadar et al., 2016)
Alpinia calcarata	TiO_2	60–130	Spherical	UV, FTIR and SEM	Anti-inflammatory & Antibacterial activity	(Pratheema, 2018)
Amylum	TiO_2	9	Spherical	XRD, SEM, BET and NAA	Photocatalytic activity	(Muniandy et al., 2017)
Anisomeles malabarica	TiO_2	18	-	XRD	-	(P. Saravanan et al., 2016)
Annona squamosa	TiO_2	23	Spherical	XRD, TEM, SEM and EDS	-	(S. Roopan et al., 2012)
Arnicae anthodium	TiO_2	30	-	UV, TXRF, FTIR, XRD and SEM-EDS	-	(R Dobrucka, 2017)
Azadirachta indica	TiO_2	15–50	Spherical	SEM, TEM, FTIR and XRD	Antibacterial activity	(Thakur et al., 2019)
Calotropis gigantea	TiO_2	10	Spherical	SEM, EDX and XRD	Acaricidal activity	(Marimuthu et al., 2013)
Capsicum annum	TiO_2	90–104	-	AFM, SEM, TEM, XRD, UV and FTIR	Antimicrobial activity	(Abdul jalill, 2018)
Cassia fistula	TiO_2	-	Spherical	UV, FTIR, XRD, SEM, EDAX and TGA	Antibacterial activity	(Swathi et al., 2014)
Catharanthus roseus	TiO_2	65	Irregular	XRD, FTIR, SEM and AFM	Antiparasitic activity	(Velayutham et al., 2012)
Cicer arietinum	TiO_2	14	Spherical	TEM, XRD, UV and TGA	Lithium ion batteries	(Kashale et al., 2016)
Cinnamomum tamala	TiO_2	23	Irregular	XRD, FTIR, DLS, TEM, SAED and EDX	Cytotoxicity against D145 cells	(He et al., 2017)
Citrus limon	TiO_2	-	-	FTIR, SEM and EDAX	Antibacterial activity	(Farook et al., 2017)
Citrus sinensis	TiO_2	19	Tetragonal	TEM, XRD, TGA and PSA	-	(Rao et al., 2015)
Cochlospermum gossypium	TiO_2	8–13	Spherical	XRD, FTIR, SEM-EDX, TEM, HR-TEM, UV and BET	Photocatalytic degradation of organic dye	(Saranya et al., 2011)
Curcuma longa	TiO_2	23–44	Spherical	AFM, UV, XRD and SEM	Antifungal & Anti-pathogenic activity	(Abdul Jalill et al., 2016)
Cymbopogon citratus	Fe-TiO_2	7–26	Irregular	SEM-EDS, XRD, UV and TEM	Photocatalytic activity in wastewater	(Solano et al., 2019)
Cynodon dactylon	TiO_2	13–34	Irregular	XRD, FTIR, Raman and SEM	Antibacterial & Anticancer activity	(Hariharan et al., 2017)
Echinacea purpurea	TiO_2	120	Spherical	UV, SEM, TXRF and FTIR	-	(Renata Dobrucka, 2017)
Eclipta prostrata	TiO_2	50	Spherical	FTIR, XRD, AFM and FESEM	-	(Rajakumar et al., 2012)
Euphorbia prostrata	TiO_2	84	Spherical	SAED, TEM and XRD	Cell death in Leishmania donovani	(Zahir et al., 2015)
Euphorbia thymifolia	Pd-TiO_2	19–29	-	FESEM, EDS, TEM and	Dye degradation	(Maham,

				XRD		Nasrollahzadeh, et al., 2017)
Glycosmis cochinchinensis	TiO_2	40	Spherical	UV, FTIR, SEM-EDS and TEM	Photocatalytic & Antimicrobial activity	(Rosi & Kalyanasundaram, 2018)
Hibiscus rosa-sinensis	TiO_2	-	Polydispersed	XRD, SEM and FTIR	Antibacterial activity	(Sahaya et al., 2014)
Jatropha curcas	TiO_2	13	Spherical	UV, FESEM, EDS, FTIR, XRD, DLS, BET and BJH	Photocatalytic degradation of tannery wastewater	(Goutam et al., 2018)
Lagenaria siceraria	TiO_2	7	Spherical	FTIR, SEM, EDAX, TEM and XRD	Antimicrobial activity & Free radical scavenging activity	(Kalyanasundaram & Prakash, 2015)
Momordica charantia	TiO_2	70	Irregular/Spherical	UV, XRD, FTIR, HRTEM, EDX, DLS and Zeta-potential	Antimalarial activity	(Gandhi et al., 2018)
Morinda citrifolia	TiO_2	15–19	Spherical	EDAX, FTIR, SEM and XRD	Antimicrobial activity	(Sundrarajan et al., 2017)
Moringa Oleifera	TiO_2	100	Spherical	UV and SEM	Wound-healing activity	(Sivaranjani & Philominathan, 2016)
Nerium Oleander	TiO_2	7	Spherical	FTIR, FESEM, EDX, XRD and UV	Photocatalytic activity	(Deshmukh et al., 2018)
Nyctanthes arbor-tristis	TiO_2	100–150	Spherical	XRD, PSA and SEM	-	(Sundrarajan & Gowri, 2011)
Ocimum basilicum	TiO_2	32	Irregular/Spherical	XRD, PSA, SEM and FTIR	-	(Alapati & Kantheti, 2018)
Peltophorum pterocarpum	TiO_2	20–80	-	XRD, HR-TEM and XANES	-	(S. Saravanan et al., 2016)
Phyllanthus niruri	TiO_2	32	Spherical	XRD, FTIR, TEM and UV	Photocatalytic activity	(Shanavas et al., 2019)
Piper betle	TiO_2	7	Spherical	FTIR, UV, XRD and SEM	Antibacterial activity	(Hunagund et al., 2016)
Pithecellobium dulce	TiO_2	6	Spherical	FTIR, SEM, EDAX, TEM and XRD	Antimicrobial activity & Free radical scavenging activity	(Kalyanasundaram & Prakash, 2015)
Psidium guajava	TiO_2	33	Spherical	XRD, FTIR, FESEM and EDX	Antibacterial & Antioxidant activity	(Santhoshkumar et al., 2014)
Punica granatum	TiO_2	75–90	-	SEM and FTIR	Pharmaceuticals	(Dubey & Singh, 2019)
Senna auriculata	TiO_2	38	Spherical	UV, FTIR, FSEM and XRD	-	(Valli & Geetha, 2015)
Sesbania grandiflora	TiO_2	43–56	Triangular, Cubic and Spherical	FTIR, SEM-EDX, UV and XRD	Toxicity in zebrafish embryos	(Srinivasan et al., 2019)
Solanum trilobatum	TiO_2	-	-	-	Toxic activity	(Vadlapudi & Behara, 2012)
Taraxacum officinale	TiO_2	-	Rod	XRD, SEM and TEM	Photocatalytic activity	(Bao et al., 2012)
Trigonella foenum-graecum	TiO_2	20–90	Spherical	FTIR, UV, XRD, HR-TEM and HR-SEM	Antimicrobial activity	(Subhapriya & Gomathipriya, 2018)
Vigna radiata	TiO_2	-	Oval	FTIR and SEM	Anti-bacterial activity	(Chatterjee et al., 2016)
Vigna unguiculata	TiO_2	-	Oval	FTIR and SEM	Antimicrobial & Cytotoxic Effects	(Chatterjee et al., 2017)

Table S7. The Plant-Mediated Synthesis of Iron & Iron Oxide Nanoparticles.

Plant Species	Nanoparticle	Particle Size (nm)	Morphology	Characterisation	Application	Reference
Abelmoschus esculentus	Fe	24–34	Spherical	XRD, UV, FTIR, FESEM and TEM	Antibacterial activity	(Pande et al., 2015)
Acacia nilotica	Fe	229	Irregular	FTIR, UV, DLS, TEM, SEM, EDS and XRD	Catalytic, adsorption and antibacterial activity	(Da'na et al., 2018)
Ailanthus excelsa	α-Fe_2O_3	40	Spherical	FTIR, XRD, SEM and UV	Mortality efficacy on green peach Aphid	(Asoufi et al., 2018)
Albizia lebbeck	Fe	-	-	XRD and FTIR	Dye degradation	(Bharadwaj et al., 2016)
Amaranthus dubius	Fe	43–200	Spherical	UV, FTIR, SEM, XRD, PSD and Zeta potential	Photocatalytic activity	(Harshiny et al., 2015)
Apium graveolens	Fe_2O_3	20–99	Irregular, Cubic and Hexagonal	XRD, FESEM and UV	Antibacterial activity	(Aziz & Urabe, 2019)
Artemisia vulgaris	Fe_3O_4	30	Spherical	TEM, PSA, XRD, FTIR, VSM and TGA	Dye degradation	(Beheshtkhoo et al., 2018)
Artocarpus altilis	Fe_2O_3	36–44	Spherical	UV, FTIR and SEM	Antibacterial, biofilm inhibition and dye degradation activities	(Kanchana & Zantye, 2018)
Avicennia marina	FeO	30–100	Cubic	UV, FTIR, SEM, AFM and XRD	Dye degradation	(Karpagavinayagam & Vedhi, 2019)
Azadirachta indica	Fe	98-500	Spherical	SEM, EDS, UV and FTIR	Antibacterial activity	(Devatha et al., 2018)
Camellia sinensis	Fe_2O_3	23–43	Irregular, Cubic and Hexagonal	XRD, FESEM and UV	Antibacterial activity	(Aziz & Urabe, 2019)
Caricaya papaya	Fe_3O_4	33	Capsule-like	FTIR, UV, XRD, SEM and EDS	-	(Latha & Gowri, 2014)
Carum carvi	Fe_2O_3	300	Spherical	UV, XRD, and SEM	Anti-cancer drug adsorption	(Izadi et al., 2018)
Centella asiatica	Fe_2O_3	10–50	Spherical	FTIR, XRD, EDX, VSM, SEM and TEM	Acute oral toxicity studies	(Pravallika et al., 2019)
Ceratonia siliqua	Fe_2O_3	7	Spherical	TEM, SEM, EDX, XRD, BET, FESEM and Raman	AMX degradation	(Demirezen, Yıldız & Yılmaz, 2019)
Citrus maxima	Fe	10–100	Irregular	TEM, EDS, XPS, FTIR, DLS and Zeta potential	-	(Wei et al., 2016)
Citrus reticulum	α-Fe_2O_3	20–63	Spherical	UV, TGA, XRD, FTIR, SEM and TEM	Dye degradation	(H. R. Ali et al., 2017)
Coffea arabica	Fe	50–100	Spherical	UV, XRD and SEM	-	(Pattanayak & Nayak, 2012)
Couroupita guianensis	Fe_3O_4	17	Spherical and Polydispersed	UV, FTIR, XPS, DLS, XRD, AFM, HRTEM, VSM and Zeta potential	Cytotoxicity and antibacterial activity	(Sathishkumar et al., 2018)
Curcuma longa	Fe_3O_4	338.2–488.1	Cubic	UV, SEM-EDX, XRD and FTIR	Wastewater treatment	(Herlekar et al., 2015)
Cydonia oblonga Miller	99mTc-labeled-Fe_3O_4	50	Spherical	FESEM, DLS, XRD, VSM and FTIR	Evaluation of their cytotoxicity and biodistribution in rats	(Rahmani et al., 2019)
Daphne mezereum	Fe_2O_3	9.2	Spherical	TEM, PSA, FTIR, XRD, VSM	Dye degradation	(Beheshtkhoo et al., 2018)
Datura inoxia	Fe_3O_4	7–14	Spherical	UV, FTIR, XRD and TEM	-	(Das et al., 2014)
Dodonaea viscose	Fe	27	Spherical	UV, XRD, AFM and HRTEM	Antibacterial activity	(S. C.G. Kiruba Daniel et al., 2013)
Emblica officinalis	Fe	22.6	Spherical	FTIR, XRD, UV, SEM and TEM	Remediation of lead-contaminated	(Kumar et al., 2015)

					water	
Eriobotrya japonica	Fe_3O_4	100	Irregular	DLS, XRD, FTIR and SEM/EDX	Heterogeneous Fenton-like catalyst for the degradation of Basic Red 46	(Önal et al., 2017)
Eucalyptus	Fe	20–80	Spherical	SEM, EDS, XRD and FTIR	Treatment of eutrophic wastewater	(T. Wang et al., 2014)
Ficus carica	Fe_2O_3	9	Spherical	EDX, TEM, UV, FTIR and DLS	-	(Demirezen, Yıldız, Yılmaz, et al., 2019)
Gardenia jasminoides	Fe	32	Hexagonal	TGA, FTIR, TEM, SEM, AFM and XRD	Antibacterial activity	(Naseem & Farrukh, 2015)
Gardenia resinifera	α-Fe_2O_3	5	Spherical	XRD, EDS and TEM	Hyperthermia	(Karade et al., 2019)
Glycine max	Fe_3O_4	8	Spherical	XPS, FTIR, SEM, TEM, SAED and XRD	-	(Cai et al., 2010)
Glycosmis mauritiana	Fe_2O_3	58–79	Spherical	UV, DLS, XRD, FTIR, SEM and TEM	Antibacterial activity	(Amutha & Sridhar, 2015)
Hordeum vulgare	Fe_3O_4	30	-	TEM, SAED, EELS, XPS and DLS	-	(Makarov et al., 2014)
Hydrangea paniculata	Fe_2O_3	56	Spherical	XRD, FTIR, SEM and TEM	Enhancement of Flax plant	(Karunakaran et al., 2017)
Kappaphycus alvarezii	Fe_2O_3	10–30	Spherical	XRD, EDX and FTIR	Photocatalytic and Antibacterial activity	(Arularasu et al., 2018)
Lagenaria siceraria	Fe_3O_4	30–100	Cubic	UV, SEM, EDX, Zeta sizer and FTIR	Antimicrobial activity	(Kanagasubbulakshmi & Kadirvelu, 2017)
Lawsonia inermis	Fe_2O_3	150–200	Spherical	UV, FTIR, SEM and EDX	Antibacterial activity	(Chauhan & Upadhyay, 2019)
Luma apiculata	Fe_xO_y	7.5-13.5	Irregular	UV, FTIR, DLS, Zeta potential, SEM-EDX and Raman	Photocatalytic activity	(Salgado et al., 2019)
Magnolia champaca	Fe	96–110	Spherical	UV, SEM, EDX and FTIR	Wastewater treatment	(Devatha et al., 2016)
Mangifera indica	Fe	100–150	Spherical	UV, SEM, EDX and FTIR	Wastewater treatment	(Devatha et al., 2016)
Mansoa alliacea	β-Fe_2O_3	18.22	Hexagonal	XRD, FTIR, UV and TGA	-	(Prasad, 2016)
Melaleuca nesophila	Fe	40–60	Spherical	SEM, EDS, XRD and FTIR	Dye degradation	(Z. Wang et al., 2014)
Mimosa pudica	Fe_2O_3	67	Spherical	UV, FTIR, XRD, SEM, PDA and VSM	-	(Niraimathee et al., 2016)
Moringa oleifera	Fe	3.4–7.4	Spherical	UV, XRD, FTIR, TEM	Removal of Nitrate from water and antibacterial activity	(Katata-Seru et al., 2018)
Murraya koenigii	Fe	-	-	UV, SEM, EDX and FTIR	Antibacterial activity	(Subha et al., 2017)
Musa acuminata	Fe_3O_4	72	Spherical	XRD, FTIR, TEM, UV and PSA	-	(Kale, Barwar, Kane & Bhatt, 2018)
Musa ornata	Fe	43.69	-	UV, FTIR, XRD, AFM and PSA	Antibacterial activity	(Saranya et al., 2017)
Myrtus communis	Fe	40–60	Spherical	UV, FTIR, XRD, SEM and TEM	Reducing excessive iron in mice	(Eslami et al., 2018)
Nephelium lappaceum	Fe_3O_4	200	Spinel	XRD, Raman, FTIR and TEM	-	(Yuvakkumar & Hong, 2014)
Nephrolepis auriculata	Fe	40–70	Spherical	TEM, XRD, EDS, XPS and FTIR	Cr(IV) removal	(Yi et al., 2019)
Ocimum sanctum	Fe_2O_3	20	Irregular	FTIR, XRD, SEM and TEM	Microscopic studies	(Balamurugan et al., 2014)
Oolong tea	Fe	40–50	Spherical	SEM, UV, BET, EDS and XRD	Degradation of malachite green dye	(Huang et al., 2014)
Passiflora foetida	Fe_2O_3	10–16	Spherical	UV, XRD, SEM and FTIR	Antibacterial activity	(Suganya, D., Rajan, M. R. and Ramesh., 2016)
Passifora tripartite	Fe_3O_4	22.3	Spherical	TEM, DLS, FTIR, UV and	2-arylbenzimidazole	(Kumar et al., 2014)

				XRD	fabrication	
Phragmites australis	Fe_xO_y	24.4-68.1	Irregular	UV, FTIR, DLS, Zeta potential, SEM-EDX and Raman	Photocatalytic activity	(Salgado et al., 2019)
Phyllanthus Niruri	Fe_3O_4	10	Cubic	IR, UV, TEM, SEM and XRD	Antimicrobial activity	(Viju Kumar & Prem, 2018)
Piliostigma thonningii	Fe	20,000	Irregular clusters	XRD and SEM	Antibacterial activity	(Igwe & Nwamezie, 2018)
Platanus orientalis	Fe_2O_3	78–80	Spherical	XRD, SEM, TEM, UV, FTIR, EDX and DLS	Antifungal activity	(Devi et al., 2019)
Prunus serotina	Fe	12	Spherical	DLS, TEM, XRD and FTIR	-	(Murgueitio et al., 2016)
Psidium guajava	Fe_3O_4	13–23	-	UV, FTIR, SEM and TEM	Doxorubicin drug loading	(Moustafa & Din, 2017)
Punica granatum	Fe	10	-	UV	Dye degradation	(Ozkan et al., 2018)
Ricinus communis	Fe	10–35	Irregular	UV, FTIR, XRD, XPS, SEM, EDX and TEM	Catalytic effects in transesterification of castor oil	(Rengasamy et al., 2016)
Rosemarinus officinalis	Fe	50	Spherical	DLS, FESEM, XRD, TEM, Raman and FTIR	Cytotoxic activity	(Farshchi et al., 2018)
Rumex acetosa	Fe_3O_4	10–40	-	TEM, SAED, EELS, XPS and DLS	-	(Makarov et al., 2014)
Sageretia thea	Fe_2O_3	30	Tetragonal	XRD, FTIR, Raman, EDS, HR-SEM, HR-TEM and SAED	Pharmacognostic properties	(Khalil, Ovais, Ullah, Ali, Khan Shinwari, et al., 2017)
Salvia officinalis	Fe	5–25	Spherical	SEM, XPS, FTIR and XRD	Dye degradation	(Wang et al., 2015)
Simarauba glauca	Fe_2O_3	43–48	Spherical	UV, FTIR and SEM	Antibacterial, biofilm inhibition and dye degradation activities	(Kanchana & Zantye, 2018)
Sorghum bran	Fe	50	Irregular	FESEM, HR-TEM, UV, XRD, EDS and Zeta potential	Dye degradation	(Njagi et al., 2011)
Spinacia oleracea	Fe	-	-	UV	Wastewater treatment	(Turakhia et al., 2018)
Stevia rebaudiana	Fe_2O_3	25	Spherical	XRD, FESEM, HRTEM, TGA, XPS, VSM and Zeta potential	Antioxidant activity	(Khatami et al., 2019)
Syzygium aromaticum	FeO	23–29	-	UV, FTIR, DLS, Zeta potential, XRD and PSA	Cytotoxic activity	(Thenmozhi et al., 2019)
Terminalia chebula	Fe	80	Irregular	UV, XRD, FTIR, EDS and TEM	-	(Mohan Kumar et al., 2013)
Trachyspermum ammi	Fe	50–90	-	UV and DLS	-	(Rani et al., 2016)
Vaccinium floribundum	Fe	5–10	Spherical	TEM, XRD and FTIR	Removal of petroleum oil from contaminated water and soil	(Murgueitio et al., 2018)
Vitex negundo	Fe_2O_3	20–22	Spherical and Hexagonal	UV, XRD, SEM and HR-TEM	-	(Karnan et al., 2018)

Table S8. The Plant-Mediated Synthesis of Cobalt and Cobalt Oxide Nanoparticles.

Plant Species	Nanoparticle	Particle Size (nm)	Morphology	Characterisation	Application	Reference
Apium graveolens	Co_3O_4	21–72	Irregular	FESEM, XRD and UV	Antibacterial activity	(Urabe & Aziz, 2019)
Aspalathus linearis	Co_3O_4	2–7	Spherical	HRTEM, EDS, XRD, ATR-FTIR, Raman, XPS		(Diallo et al., 2015)
Azadirachta indica	Co_3O_4	1–7	Quasi-spherical	XRD, HRTEM, EDX, DRS, PL, Raman and VSM	Photocatalytic and antimicrobial activity	(Sivachidambaram et al., 2017)
Calotropis gigantea	Co_3O_4	50–60	Spherical	XRD, UV, SEM, TEM and EDX	Photocatalytic and electrocatalytic activity	(J. K. Sharma et al., 2015)
Calotropis procera	Co_3O_4	10	Spherical	XRD, DSC, TEM, EDX, FTIR and UV	Ecotoxic activity	(Dubey et al., 2018)
Camellia sinensis	Co_3O_4	21–72	Irregular	FESEM, XRD and UV	Antibacterial activity	(Urabe & Aziz, 2019)
Chromolaena odorata	Co	20–49	Irregular, Cubic and Hexagonal	UV, FTIR, SEM and XRD	Antibacterial activity	(Igwe, 2018)
Conocarpus erectus	Co	20–60	Spherical	SEM and XRD		(Ahmed, 2016)
Euphorbia heterophylla	Co_3O_4	69.75	Spherical	FTIR, UV, PSA, XRD and DRS	Photocatalytic activity	(Dewi et al., 2019)
Geranium wallichiamum	CoO	21	Agglomerated	SEM, XRD, DLS, FTIR, EDX and Raman	Antibacterial, antioxidant, cytotoxic and enzyme inhibition properties	(Iqbal, Abbasi, Batool, et al., 2019)
Gingko biloba	Co_3O_4	30–100	Irregular	XRD, TEM and SEM	Enhanced electrocatalysis	(Han et al., 2015)
Helianthus annus	Co_3O_4	200–500	Plate-shaped	XRD, TGA, SEM and PSA	Photocatalytic activity	(Nomura et al., 2019)
Hibiscus rosa-sinensis	Co_3O_4	40.05–61.32	Cubical spinel	XRD, FESEM, EDX, PL, FTIR and UV	Antibacterial and antifungal activity	(Anuradha & Raji, 2019)
Mangifera indica	Co	25–40	Irregular, Cubic and Pentagonal	UV, XRD, FTIR and SEM	Detection of Manganese (II) Ions in industrial wastewater	(Okwunodulu et al., 2019)
Manihot esculenta crantz	Co_3O_4	-	Octahedral	XRD, SEM, EDAX, TEM, TGA, FTIR and VSM		(Ikhuoria et al., 2018)
Moringa oleifera	Co_3O_4	20–50	Spinel cubic	XRD, EDS, EDX, SAED, HRTEM	Electrochemical electrode for supercapacitors	(Matinise et al., 2018)
Nerium indicum	Co	20–60	Spherical	SEM and XRD		(Ahmed, 2016)
Ocimum sanctum	Co	-	Spherical	UV, FTIR, SEM, EDAX and XRD	Antimicrobial activities	(Kuchekar et al., 2018)
Piper longum	Co	-		CV	Sensing organic compounds	(Ranaei Siadat, 2015)
Punica granatum	Co_3O_4	40–80	Spherical	XRD, SEM, EDX, AFM, FTIR and UV	Photocatalytic activity	(Bibi, Nazar, et al., 2017)
Raphanus sativus	Co	80	Spherical	UV, FTIR and SEM	Antibacterial and cytotoxic activity	(Koyyati et al., 2016)
Sageretia thea	Co_3O_4	20.03	Cubic	XRD, FTIR, Raman, HRTEM, HRSEM, SAED and EDS	Antibacterial, cytotoxic and antileishmanial activity	(Khalil, Ovais, Ullah, Ali, Shinwari, et al., 2017)
Sechinum edule	Co_3O_4	5.8–38.1	Irregular	XRD, FTIR, AFM, SEM, TEM and VSM	Electrochemical H2O2 sensing	(Das & Golder, 2017)
Taraxacum offici-	Co_2O_3	50–100	Spherical	UV, FTIR, DLS, SEM	Dye degradation	(Rasheed et al., 2019)

nale				and TEM		
Vitis vinifera	Co_3O_4	10–20	Spherical rods	XRD, FTIR, Raman, TEM, SAED, EDX, DRS, PL and VSM	Catalytic, photocatalytic and antibacterial activity	(Kombaiah et al., 2018)

Table S9. The Plant-Mediated Synthesis of Nickel and Nickel Oxide Nanoparticles.

Plant Species	Nanoparticle	Particle Size (nm)	Morphology	Characterisation	Application	Reference
Aegle marmelos	NiO	8–10	Spherical	HRSEM, HRTEM, XRD and FTIR	Cytotoxic, antibacterial and photocatalytic activity	(Ezhilarasi et al., 2018)
Agathosma betulina	NiO	15–55	Cubic	TEM, EDS, XRD, FTIR, Raman and PL	Photodiode activity	(Thema et al., 2016)
Ageratum conyzoides	NiO	8–15	Cubic	UV, FTIR, XRD and TEM	Catalytic activity	(Wardani et al., 2019)
Aloe vera	NiO	50–70	Rod-like	SEM, XRD and FTIR	Catalytic activity	(Juibari & Eslami, 2019)
Azadirachta indica	Ni & NiO	17–70	Spherical	TEM, SEM, XRD and AMS	Cytotoxic activity	(Mariam et al., 2014)
Callistemon viminalis	NiO	20–35	-	UV, Raman, SEM, XPS and XRD	Pseudo-capacitors	(Sone et al., 2016)
Calotropis gigantea	Ni & NiO	20–60	-	UV, FTIR and XRD	Catalytic and antimicrobial activity	(Din et al., 2018)
Camellia sinensis	Ni	43.87–48.76	Spherical	SEM, EDX and XRD	Photocatalytic activity	(Bibi, Kamal, et al., 2017)
Coriandrum sativum	Ni	30.71	-	UV, FTIR and XRD	-	(Vasudeo & Pramod, 2016)
Desmodium gangeticum	Ni	-	Spherical	UV, XRD, FTIR, VSM and Zeta potential	Biological activities	(Sudhasree et al., 2015)
Dioscorea orangeana	Ni	2–3	Spherical	UV, XRD, SEM and EDAX	Antimicrobial activity	(Helen & Rani, 2015)
Geranium wallichianum	NiO	21	Spherical	XRD, SEM, TEM, FTIR, EDS, Raman, DLS and UV	Biological activities	(Abbasi et al., 2019)
Hibiscus rosa-sinensis	Ni	10–200	Spherical	SEM, TEM and XRD	-	(Kar & Ray, 2014)
Medicago sativa	Ni	1–6	Spherical	TEM, XRD, FTIR and XPS	-	(Chen et al., 2014)
Monsonia burkenea	NiO	20	Spherical	FTIR, EDX, HRTEM and XRD	Photocatalytic, antibacterial and cytotoxic activity	(Kganyago et al., 2018)
Moringa oleifera	NiO	-	Spherical	XRD, FTIR, HRTEM, EDX and PL	Cytotoxic activity	(Ezhilarasi et al., 2016)
Nephelium lappaceum	NiO	50	Irregular	TEM, SEM, PSA and XRD	Antibacterial activity	(Yuvakkumar et al., 2014)
Ocimum sanctum	Ni	15–36	Spherical to polyhedral	FTIR, UV, XRD, SEM and TEM	Dye and pollutant adsorption	(Pandian et al., 2015)
Physalis angulata	NiO	64.13	Spherical	FTIR, Raman, XRD, UV, PSA, SEM-EDS and TEM-SAED	-	(Sulaiman & Yulizar, 2018)
Piper betle	Ni	31	Spherical	UV, FTIR, TEM, EDX, PSA, RSM and XRD	-	(Kale, Barwar, Kane & Contractor, 2018)
Psidium guajava	Ni & NiO	17–70	Spherical	TEM, SEM, XRD and AMS	Cytotoxic activity	(Mariam et al., 2014)
Rhamnus virgata	NiO	24	Spherical	UV, XRD, FTIR, SEM, EDS, TEM, Raman and DLS	Biological activities	(Iqbal, Abbasi, Mahmood, et al., 2019)
Rheum turkestanicum	NiO	12–15	Cubic	FESEM, EDX, FTIR and XRD	Photocatalytic and cytotoxic activity	(Saheb et al., 2019)
Tamarix serotine	NiO	10–14	Spherical	XRD, FTIR, UV, TEM, VSM and BET	Catalytic activity	(Nasseri et al., 2016)
Taxus brevifolia	Ni	22	Hexagonal	UV, SEM, FESEM, XRD, FTIR and TEM	Antibacterial and anticancer activity	(Sarli et al., 2018)

Table S10. The Plant-Mediated Synthesis of Copper and Copper Oxide Nanoparticles.

Plant Species	Nanoparticle	Particle Size (nm)	Morphology	Characterisation	Application	Reference
Abutilon indicum	CuO	16.78	Hexagonal	XRD, EDX, SEM and UV	Antimicrobial, antioxidant and photocatalytic activities	(Ijaz et al., 2017)
Acanthospermum hispidum	CuO	5–25	Spherical	FESEM, EDX, TEM, XRD, FTIR and PL	Biological activities	(Pansambal, Deshmukh, et al., 2017)
Aglaia elaeagnoidea	CuO	20–45	Spherical	FESEM, XRD, EDX, TEM and TGA-DSC	Catalytic and recyclability properties	(Manjari et al., 2017)
Albizia lebbeck	CuO	100	Spherical	UV, SEM, TEM, EDS and XRD	-	(Jayakumarai et al., 2015)
Alchornea laxiflora	Cu	3.29	Spherical	UV, HRTEM, EDX and FTIR	Oxidative desulphurisation of model oil	(Olajire et al., 2018)
Allium cepa	Cu	21–23	Spherical	UV, AFM, SEM and EDX	Enhancement of plant growth	(Mansi P. et al., 2017)
Aloe barbadensis	Cu	80–120	Spherical & Cubic	UV, FTIR, XRD and SEM	Photocatalytic activity	(Batool, 2018a)
Aloe vera	Cu	251.1	-	TEM, UV, Zeta potential	Bioleaching	(Pawlowska & Sadowski, 2017)
Anthemis nobilis	CuO	18.02–61.29	Rectangular	SEM, EDS, XRD, FTIR and UV	Catalytic activity	(Nasrollahzadeh, Mohammad Sajadi, et al., 2015)
Arbutus unedo	CuO	33	Spherical	XRD, FTIR, UV, EDS and TEM	Cytotoxic activity	(Yu et al., 2018)
Asparagus adscendens	Cu	10–15	Spherical	UV, FTIR and HRTEM	Antimicrobial activity	(Thakur et al., 2018)
Azadirachta indica	Cu	48–73	Cubic	SEM, UV, FTIR, TEM, EDS and XRD	-	(Nagar & Devra, 2018)
Bauhinia tomentosa	CuO	22–40	Spherical	XRD, UV, TEM, EDX and FTIR	Antibacterial activity	(Sharmila et al., 2018)
Calotropis procera	Cu	15	Spherical	TEM, XRD, EDAX and FTIR	Cytotoxic activity	(Harne et al., 2012)
Calotropis gigantea	CuO	20	Spherical	FESEM, EDX, TEM, XRD and FTIR	Dye-sensitised solar cells application	(Jitendra Kumar Sharma et al., 2015)
Camellia sinensis	Cu	50–100	-	TEM, UV and XRD	-	(Mohindru & Garg, 2017)
Capparis spinosa	Cu	17–41	Spherical	UV, FTIR, SEM and EDX	Antibacterial activity	(Ebrahimi et al., 2017)
Capparis zeylanica	Cu	50–100	Cubic	UV, FTIR, XRD, SEM, EDX and TEM	Antimicrobial activity	(Saranyaadevi et al., 2014)
Carica papaya	Cu	20	Spherical	UV, FTIR, XRD, SEM and TEM	-	(Suresh et al., 2014)
Cassia auriculata	Cu	38.1–43.5	Spherical	FESEM, XRD, UV and FTIR	-	(Mohan et al., 2017)
Citrus limon	Cu	60–100	Spherical	UV, FTIR, XRD, SEM and TEM	Antimicrobial activity	(Jayandran et al., 2015)
Citrus medica Linn	Cu	10–60	-	UV, DLS and XRD	Antimicrobial activity	(Shende et al., 2015)
Citrus sinensis	Cu	2	Spherical	UV	Antifungal activity	(Kale et al., 2017)
Coffea	CuO	100	Spherical	SEM, XRD, UV and FTIR	Antibacterial activity	(Sutradhar et al., 2014)
Cordia sebestena	CuO	20–35	Spherical	UV, FTIR, FESEM-EDX, TEM, SAED, DLS and Zeta potential	Photocatalytic and antibacterial activity	(Prakash et al., 2018)
Curcuma longa	Cu	5–20	Spherical	TEM, FESEM, XRD,	Antimicrobial activ-	(Jayarambabu et al., 2020)

				FTIR, UV and EDS	ity	
Cymbopogon	Cu/CuO	5.67–9.10	Spherical	UV, TEM, XRD and EDX	Photocatalytic activity	(Tu, 2019)
Cynomorium coccineum	Cu	14.2	Spherical	SEM, EDX, XRD, TGA and FTIR	Photocatalytic activity	(Sebeia et al., 2020)
Drypetes sepiaria	CuO	18.77	Spherical	UV, XRD, FTIR and TEM	Photocatalytic activity	(Narasaiah, Mandal, et al., 2017)
Duranta erecta	Cu	70	Spherical	UV, FTIR, FESEM, EDX, TGA and XRD	Photocatalytic activity	(Ismail et al., 2019)
Eucalyptus camaldulensis	CuO	40	Rod	SEM and UV	Antimicrobial activity	(Shanan et al., 2018)
Eclipta prostrata	Cu	23–57	Cubic	SEM, TEM, EDX, XRD, FTIR, SAED and UV	Antioxidant and cytotoxic activities	(Chung et al., 2017)
Euphorbia chamaesyce	CuO	36–40	Irregular	FESEM, EDX, FTIR, TEM and UV	Catalytic activity	(Maham, Sajadi, et al., 2017)
Euphorbia esula	Cu	20–110	Spherical	UV, XRD and TEM	Catalytic activity	(Nasrollahzadeh et al., 2014)
Ferulago angulata	CuO	32–36	Spherical	XRD, FTIR and FESEM	Photocatalytic activity	(Shayegan Mehr et al., 2018)
Ficus religiosa	CuO	577	Spherical	FESEM, FTIR, DLS and XRD	Cytotoxic activity	(Sankar et al., 2014)
Ginkgo biloba	Cu	15–20	Spherical	TEM, EDS, FTIR and UV	Catalytic activity	(Nasrollahzadeh & Mohammad Sajadi, 2015)
Gloriosa superba	CuO	5–10	Spherical	UV, XRD, SEM and TEM	Antibacterial activity	(Naika et al., 2015)
Gum karaya	CuO	4.8–7.8	Irregular	SEM, XRD, FTIR, TEM and XPS	Antibacterial activity	(Padil & Černík, 2013)
Hibiscus rosa-sinensis	CuO	45–80	Spherical	UV, FTIR, XRD, SEM and EDX	Antibacterial activity	(Rajendran et al., 2018)
Ixiro coccinea	CuO	80–110	Irregular	UV, SEM, TEM and FTIR	-	(Vishveshvar et al., 2018)
Lawsonia inermis	Cu	43	Spherical	UV, XRD, FTIR, HRTEM, HRSEM and EDX	Nano bio-composites	(Cheirmadurai et al., 2014)
Macrocystis pyrifera	Cu	102.4	Spherical	SEM, TEM, EDS and Zeta potential	-	(Araya-Castro et al., 2017)
Malus domestica	CuO	34	Cubic	SEM, FTIR, XRD and UV	Antifungal activity	(Choudhary et al., 2018)
Malva sylvestris	CuO	5–30	Spherical	FTIR, SEM, XRD and UV	Antibacterial activity	(Awwad et al., 2015)
Mimusops elengi	Cu	-	Rod & Spherical	UV, FTIR, SEM and XRD	Biological activities	(Iyer & Lilian R, 2019)
Moringa oliefera	Cu	-	-	UV and XRD	Enhancement of plant growth	(Sabir et al., 2018)
Murraya koenigii	Cu	-	Spherical	UV, FTIR and SEM	-	(Ashtaputrey et al., 2017)
Musa paradisica	CuO	60	Spherical	XRD, EDX, FESEM and FTIR	Photocatalytic activity	(Aminuzzaman et al., 2017)
Nerium odorum	Cu	139.2	-	UV, PSA, FTIR and TEM	Antimicrobial activity	(Srivastava & Dwivedi, 2018)
Nerium oleander	Cu	-	-	UV and FTIR	Antibacterial activity	(Gopinath et al., 2014)
Ocimum basilicum	CuO	70	Spherical	UV, FTIR and SEM	Antibacterial activity	(Altikatoglu et al., 2017)
Ocimum sanctum	Cu	77	-	XRD and FTIR	-	(Kulkarni & Kulkarni, 2013)
Ocimum tenuiflorum	CuO	-	Irregular	FTIR, SEM, XRD and EDX	-	(Sumitha et al., 2016)
Olea europaea	CuO	20–50	Spherical	XRD, FTIR, SEM and TEM	Toxic activity	(Sulaiman et al., 2017)
Passiflora foetida	Cu	24.54	Spherical	UV, FTIR, SEM,	Antimicrobial activ-	(Fatma et al., 2017)

				XRD, EDX and DLS	ity	
Pelargonium graveolens	Cu	164.7	-	TEM, UV, Zeta potential	Bioleaching	(Pawlowska & Sadowski, 2017)
Phaseolus vulgaris	CuO	27	Spherical	XRD, FTIR, XPS, Raman, DLS, TEM, SAED, SEM and EDX	Cytotoxic activity	(Nagajyothi et al., 2017)
Pheonix dactylifera	CuO	22–28	Spherical	UV, FTIR, XRD, SEM and EDAX	-	(Berra et al., 2018)
Phyllanthus embilica	Cu	15–30	Flakes	UV, FTIR, XRD, SEM and EDAX	Antimicrobial activity	(Caroling et al., 2015)
Plantago asiatica	Cu	7–35	Spherical	FTIR, UV, TEM and XRD	Cyanation of aldehydes	(Nasrollahzadeh et al., 2017)
Polygonum minus	Cu	20–30	Spherical	UV, XRD, FTIR, DLS, FESEM-EDX, Zeta potential and TGA	Photocatalytic and antibacterial activity	(Ullah et al., 2019)
Psidium guajava	CuO	2–6	Spherical	UV, FTIR, FESEM, TEM, EDX and XRD	Photocatalytic activity	(Singh et al., 2019)
Pterospermum acerifolium	CuO	100–250	Oval	UV, FESEM, EDX, FTIR, XPS, Zeta potential and DLS	Toxicity towards *Daphnia magna*	(Saif et al., 2016)
Punica granatum	CuO	40	Spherical	FTIR, SEM, UV and XRD	Effect on green peach Aphid	(Ghidan et al., 2016)
Rheum palmatum	CuO	10–20	Spherical	FTIR, UV, XRD, SEM and EDX	Catalytic activity	(Bordbar et al., 2017)
Rosa canina	CuO	15–25	Spherical	XRD, FTIR, FESEM, EDS, TEM and UV	Catalytic activity	(Hemmati et al., 2018)
Saraca indica	CuO	40–70	Spherical	UV, FTIR, XRD, EDX, XPS, SEM, TEM and HRTEM	-	(Prasad et al., 2017)
Solanum tuberosum	Cu	5–40	Spherical	UV, XRD, FTIR, SEM and TEM	-	(Suresh et al., 2014)
Stachys lavandulifolia	Cu/Cu_2O	80	Spherical	UV, TEM, XRD and FTIR	-	(Khatami et al., 2017)
Syzygium alternifolium	CuO	2–69	Spherical	FTIR, UV, XRD, AFM, SEM, TEM and DLS	Antiviral activity	(Yugandhar et al., 2018)
Syzygium aromaticum	Cu	20	Spherical	FESEM, HRTEM, EDS, Zeta potential, UV, XRD and FTIR	Antimicrobial activity	(K. M. Rajesh et al., 2018)
Tabernaemontana divaricate	CuO	48	Spherical	UV, XRD, FTIR, SEM, EDX and TEM	Antibacterial activity	(Sivaraj et al., 2014)
Terminalia arjuna	Cu	20–30	Spherical	XRD, FTIR, TGA, FESEM and EDX	Biological activity	(Yallappa et al., 2013)
Terminalia catappa	Cu/CuO/Cu_2O	21–30	Spherical	TEM, SEM, FTIR, XRD and TGA	Antibacterial activity	(Muthulakshmi et al., 2017)
Thymbra spicata	CuO	10–20	Spherical	FTIR, XRD, FESEM, TEM, EDS, WDX and UV	Catalytic activity	(Veisi et al., 2017)
Thymus vulgaris	CuO	-	-	TEM, EDS, FTIR, XRD, TGA and DTA	Catalytic activity	(Nasrollahzadeh et al., 2016)
Tinospora cardifolia	Cu	63.3	Spherical	UV, XRD, PSA, Zeta potential, FESEM, EDX, AFM, Raman and TEM	Antimicrobial activity	(P. Sharma et al., 2019)
Vitis vinifera	CuO	25–50	Spherical	UV, FTIR, XRD, EDX and SEM	-	(Demirci Gultekin et al., 2017)
Ziziphus mauritiana	CuO	20–45	Spherical	XRD, SEM, EDX and	-	(Pansambal, Gavande, et al.,

				TEM		2017)
Ziziphus spina-christi	Cu	5–20	Spherical	UV, FTIR, FESEM, TEM and XRD	Photocatalytic and antibacterial activity	(Khani et al., 2018)

Table S11. The Plant-Mediated Synthesis of Zinc and Zinc Oxide Nanoparticles.

Plant Species	Nanoparticle	Particle Size (nm)	Morphology	Characterisation	Application	Reference
Acalypha indica	ZnO	20	Spherical	XRD, PSA, SEM, TEM and SAA	Textile coating, hydrophobicity, UV resistance and antibacterial activity	(Karthik et al., 2017)
Adhatoda vasica	ZnO	85–95	Spherical & Irregular	XRD, FTIR and SEM	Catalytic activity	(Anvekar et al., 2017)
Agathosma betulina	ZnO	15.8	Quasi-spherical	TEM, EDX, XRD, FTIR and Raman	-	(Thema et al., 2015)
Albizia lebbeck	ZnO	66.25–112.87	Spherical & Irregular	UV, XRD, FTIR, Zeta potential, SEM and EDX	Antimicrobial, antioxidant and cytotoxic activities	(Umar et al., 2019)
Allium sativum	ZnO	88.67–101.59	Spherical	AFM, XRD, FTIR and UV	-	(Slman et al., 2018)
Aloe barbadensis	ZnO	25–40	Spherical	UV, FTIR, PL, SEM, TEM and XRD	-	(Sangeetha et al., 2011)
Aloe vera	ZnO	22.18	Hexagonal	XRD, SEM, UV, PL, BET and TGA	Photocatalytic and antibacterial activity	(Varghese & George, 2015)
Ananas comosus	ZnO	30–57	Hexagonal	FESEM, EDX, UV and XRD	Antibacterial activity	(Ahmad et al., 2019)
Andrographis paniculata	Zn	-	-	XRD, EDX, SEM, FTIR and UV	Antimicrobial activity	(Devasenan et al., 2016)
Anisochilus carnosus	ZnO	20–40	Spherical	PL, FTIR, XRD, TEM and FESEM	Photocatalytic and antibacterial activity	(Anbuvannan et al., 2015)
Annona muricata	ZnO	17	Semi-spherical	UV, SEM-EDS, XRD and FTIR	-	(Sierra et al., 2018)
Aspalathus linearis	ZnO	24.6–28.5	Quasi-spherical	SEM, EDS, TEM, XRD, DSC, TGA and FTIR	-	(Nethavhanani et al., 2018)
Astragalus gummifer	ZnO	50	Hexagonal	UV, XRD, TGA, FESEM and TEM	Neurotoxicity effect	(Darroudi et al., 2013)
Astragalus membranaceus	ZnO	35–38	Spherical	XRD, FTIR and TEM	-	(R. Yang et al., 2017)
Atalantia monophylla	ZnO	30	Spherical	UV, XRD, FTIR, SEM, EDX and TEM	Antimicrobial activity	(Vijayakumar et al., 2018)
Azadirachta indica	ZnO	24.7	Floral	FTIR, XRD and SEM	Antimicrobial activity	(Noorjahan et al., 2015)
Calotropis gigantea	ZnO	1.5–8.5	Spherical	UV, DLS, XRD, FTIR, SEM, EDX and AFM	Enhancement of tree growth	(Chaudhuri & Malodia, 2017)
Calotropis procera	ZnO	15–25	Spherical	XRD, DRS, TEM and FTIR	Photocatalytic activity	(Gawade et al., 2017)
Camellia sinensis	ZnO	80–120	Hexagonal	UV, FTIR, SEM and XRD	Photocatalytic activity	(Batool, 2018b)
Carica papaya	ZnO	50	Spherical	FESEM, TEM, PXRD, FTIR and UV	Photocatalytic and photovoltaic activities	(Rathnasamy et al., 2017)
Cassia auriculata	ZnO	-	Spherical	SEM, UV, PL and FTIR	-	(Ramesh et al., 2014)
Cassia densistipulata	ZnO	-	Spherical	SEM, UV and FTIR	-	(Kooluru & Sharada, 2014)
Cassia fistula	ZnO	5–15	Irregular sponge-like	UV, XRD and TEM	Photocatalytic, antioxidant and antibacterial activities	(Suresh et al., 2015)
Catharanthus roseus	ZnO	23–57	Spherical	XRD, SEM, EDX and Ra-	Antibacterial activity	(Savithramma &

				man		Bhumi, 2014)
Celosia argentea	ZnO	25	Spherical	UV, SEM, DLS, TGA, XRD and FTIR	Antimicrobial and antioxidant activities	(Vaishnav et al., 2017)
Cestrum nocturnum	Zn	10–30	Spherical	TEM and FTIR	Antibacterial activity	(N. Paul, Syed, et al., 2016)
Citrus aurantifolia	ZnO	50–200	Spherical	FESEM, PL and XRD	-	(Ain Samat & Md Nor, 2013)
Citrus limon	ZnO	21.5	Spherical	FESEM, UV, FTIR and PL	Photocatalytic activity	(Davar et al., 2015)
Citrus sinensis	ZnO	12.7–24.3	Irregular sponge-like	FTIR, XRD, HRTEM, EDX and UV	Photocatalytic activity	(Luque et al., 2018)
Cocus nucifera	ZnO	20–80	Spherical	UV, XRD, FTIR, TEM and Zeta potential	Combating microfouling	(Krupa & Vimala, 2016)
Coptidis rhizoma	ZnO	2.9–25.2	Spherical & Rod	UV, FTIR, SEM-EDX, TGA, SAED, TEM and XRD	Antibacterial, antioxidant and cytotoxic activities	(Nagajyothi et al., 2014)
Coriandrum sativum	ZnO	66	Floral	XRD, SEM, FTIR and EDX	-	(Gnanasangeetha & Saralathambavani, 2013)
Corymbia citriodora	ZnO	64	Hexagonal	SEM, EDX, XRD, UV, Raman and TGA	Photocatalytic activity	(Zheng et al., 2015)
Costus pictus	ZnO	20–80	Hexagonal	FTIR, XRD, SEM, EDX and TEM	Antimicrobial and anticancer activities	(Suresh et al., 2018)
Couroupita guianensis	ZnO	-	Hexagonal	UV, FTIR, XRD, FESEM and Zeta potential	Antimicrobial activity	(Sathishkumar et al., 2017)
Curcuma longa	ZnO	46	Rod	UV, FTIR, SEM and TEM	Antibacterial activity	(Jayandran et al., 2015)
Curcuma neilgherrensis	ZnO	-	-	UV, FTIR, SEM and XRD	Antibacterial activity	(Parthasarathy G et al., 2017)
Cymbopogon citratus	ZnO	85–95	Spherical & Irregular	XRD, FTIR and SEM	Catalytic activity	(Anvekar et al., 2017)
Duranta erecta	ZnO	-	-	UV	-	(Ravindran et al., 2016)
Eucalyptus globulus	ZnO	11.6	Spherical	XRD, FESEM, HRTEM and EDX	Photocatalytic and antioxidant activity	(Reddy & Mandal, 2017)
Garcinia mangostana	ZnO	21	Spherical	XRD, FTIR, TEM, FESEM, EDX and UV	Photocatalytic activity	(Aminuzzaman et al., 2018)
Gossypium herbaceum	ZnO	18.97	Hexagonal	XRD, EDX, SEM, FTIR and UV	-	(Sadatzadeh et al., 2018)
Hemidesmus indicus	ZnO	-	-	UV	-	(Manokari & Shekhawat, 2015)
Hibiscus rosa-sinensis	ZnO	30–35	Sponge-like	SEM and XRD	-	(Devi & Gayathri, 2014)
Hibiscus subdariffa	ZnO	16–60	Spherical	UV, FTIR, XRD, FESEM, EDX and HRTEM	Antibacterial and anti-diabetic activity	(Bala et al., 2015)
Hybanthus enneaspermus	ZnO	-	-	UV	-	(Shekhawat et al., 2014)
Ixora coccinea	ZnO	145.1	Spherical	UV, XRD, FTIR, DLS, Zeta potential, SEM and EDX	-	(Yedurkar et al., 2016)
Lantana aculeate	ZnO	12	Spherical	UV, FTIR, XRD, FESEM and HRTEM	Antifungal activity	(Narendhran & Sivaraj, 2016)
Laurus nobilis	ZnO	21.49-25.26	Spherical	UV, FTIR, XRD, EDX and SEM	-	(Fakhari et al., 2019)
Limonia acidissima	ZnO	12–53	Spherical	UV, FTIR, AFM, XRD and HRTEM	Antibacterial activity	(Patil & Taranath, 2016)
Lycopersicon esculentum	ZnO	51.6	Cubic	UV, FTIR, XRD, TEM and SEM	Antibacterial activity	(Ogunyemi et al., 2019)
Mangifera indica	ZnO	23	Semi-spherical	UV, SEM-EDS, XRD and FTIR	-	(Sierra et al., 2018)
Matricaria chamo-	ZnO	51.2	Cubic	UV, FTIR, XRD, TEM and	Antibacterial activity	(Ogunyemi et al., 2019)

milla				SEM		
Medicago sativa	ZnO	10	Spherical	XRD, EDX, SEM, TEM and FTIR	Antimicrobial activity	(Król et al., 2019)
Melia azedarach	ZnO	-	-	UV	-	(Manokari et al., 2016)
Moringa oleifera	ZnO	52	Hexagonal	UV, FTIR, SEM and XRD	Photocatalytic and antibacterial activity	(Pal et al., 2018)
Nephelium lappaceum	ZnO	25–40	Spherical	XRD, UV, DRS, FESEM and HRTEM	Photocatalytic activity	(Karnan & Selvakumar, 2016)
Nyctanthes arbor-tristis	ZnO	12–32	-	UV, FTIR, XRD, DLS and TEM	Antifungal activity	(Jamdagni et al., 2018)
Ocimum basilicum	ZnO	28.13	Floral	FTIR, XRD and SEM	Antimicrobial activity	(Noorjahan et al., 2015)
Olea europaea	ZnO	41	Cubic	UV, FTIR, XRD, TEM and SEM	Antibacterial activity	(Ogunyemi et al., 2019)
Parthenium hysterophorus	ZnO	16–45	Quasi-spherical, radial & cylindrical	FTIR, UV, SEM, EDX and TEM	Antibacterial activity	(Datta et al., 2017)
Passiflora caerulea	ZnO	70	Spherical	UV, XRD, FTIR, SEM, EDX and AFM	Antibacterial activity	(Santhoshkumar et al., 2017)
Petroselinum crispum	ZnO	40	Spherical	XRD, SEM, DLS and DRS	Antibacterial activity	(Hajiashrafi & Motakef-Kazemi, 2018)
Phyllanthus Emblica	ZnO	30	Quasi-spherical	FTIR, XRD, EDAX, PL and TEM	-	(Loganathan et al., 2018)
Phyllanthus niruri	ZnO	69.71–94.36	Spherical	FTIR, SEM and TEM	Antibacterial activity	(Noorjahan, 2019)
Physalis alkekengi	ZnO	72.5	Triangular	XRD, TEM and EDS	-	(Qu, Yuan, et al., 2011)
Plectranthus amboinicus	ZnO	20–50	Spherical & Hexagonal	UV, FTIR, TEM and XRD	Antibacterial, antibiofilm and larvicidal activity	(Vijayakumar et al., 2015)
Polygala tenuifolia	ZnO	33.03–73.48	Spherical	UV, FTIR, TGA, TEM, SEM and EDX	Antioxidant and anti-inflammatory activities	(Nagajyothi et al., 2015)
Poncirus trifoliate	ZnO	8.48–32.51	Spherical	UV, FTIR, TGA, GC-MS, XRD, SEM-EDX and TEM	Catalytic activity	(Nagajyothi et al., 2013)
Pongamia pinnata	ZnO	100	Hexagonal	XRD, UV, DLS, SEM, TEM and FTIR	Antibacterial activity	(Sundrarajan et al., 2015)
Punica granatum	ZnO	32.98-81.84	Hexagonal	UV, XRD, TEM, FESEM and FTIR	Cytotoxicity and antibacterial activities	(Mohamad Sukri et al., 2019)
Rosa canina	ZnO	50	Spherical	XRD, SEM, EDX, FTIR and DLS	Antibacterial activity	(Jafarirad et al., 2016)
Rosmarinus officinalis	ZnO	14–27	Hexagonal	XRD, FTIR, TEM and EPR	Antibacterial activity	(Stan et al., 2016)
Rubia cordifolia	ZnO	14.18	Spherical	XRD, SEM and TEM	Antibacterial activity	(Mushtaq et al., 2017)
Scadoxus multiflorus	ZnO	31	Spherical & Irregular	FTIR, XRD, Zeta potential and TEM	Antifungal, ovicidal and larvicidal activities	(Al-Dhabi & Arasu, 2018)
Sedum alfredii hance	ZnO	53.7	Pseudo-spherical	XRD, TEM and EDS	-	(Qu, Luo, et al., 2011)
Sesamum indicum	ZnO	-	-	UV	-	(Manokari et al., 2019)
Solanum nigrum	ZnO	20-30	Quasi-spherical	UV, DRS, PL, XRD, FTIR, FESEM, TEM, TG-DTA, XPS	Antibacterial activity	(Ramesh et al., 2015)
Solanum torvum	ZnO	38	Spherical	UV, XRD, FTIR, DLS, TEM, SEM and Zeta potential	Toxicity studies	(Ezealisiji et al., 2019)
Spilanthes acmella	Zn	-	Wire-like	TEM	-	(N. Paul, Khole, et al., 2016)
Spinacia oleracea	ZnO	30	-	UV, FTIR, XRD and SEM	Antimicrobial activity	(Sachudanandam et

						al., n.d.)
Tamarindus indica	ZnO	16–31	Spherical	UV, PL, XRD, FTIR, FESEM and EDX	Antibacterial and anti-fungal activities	(Elumalai et al., 2015)
Trifolium pratense	ZnO	60–70	-	UV, XRD, FTIR, SEM and EDX	Antibacterial activity	(Dobrucka & Długaszewska, 2016)
Vitex negundo	ZnO	10–130	Hexagonal	UV-DSR, XRD and DLS	Interaction with human serum albium	(Ambika & Sundrarajan, 2015)
Zingiber officinale	ZnO	30–50	Spherical	FESEM, EDX and FTIR	-	(Anand Raj & Jayalakshmy, 2015)

Table S12. The Plant-Mediated Synthesis of Palladium and Palladium Oxide Nanoparticles.

Plant Species	Nano-particle	Particle Size (nm)	Morpholo-gy	Characterisation	Application	Reference
Acacia senegal	Pd	9.1	Spherical	UV, FTIR, XRD, TEM, XPS, DLS and TGA	-	(Keerthi Devi et al., 2010)
Anacardium occi-dentale	Pd	2,5–4.5	Spherical	XRD, TEM, UV and FTIR	-	(Sheny et al., 2012)
Ananas comosus	Pd	1.71–16.14	Spherical	UV, XRD, FTIR, HRTEM and EDX	Photocatalytic activity	(Olajire & Mohammed, 2019)
Annona squamosa	Pd	100	Spherical	UV, XRD and TEM	Acaricidal, insecticidal and larvicidal activities	(S. M. Roopan et al., 2012)
Anogeissus latifolia	Pd	4.8	Spherical	UV, DLS, TEM and XRD	Antioxidant and catalytic activity	(Kora & Rastogi, 2018)
Artemisia abrotanum	Pd	20	Spherical	UV, XRD, FTIR, FESEM, TEM and EDS	Catalytic activity	(Ahmadi et al., 2018)
Aspalathus linearis	Pd & PdO	3.8–22	Spherical	UV, HRTEM, DSC, XRD, EDS, XPS and Raman	-	(Ismail et al., 2017)
Asparagus race-mosus	Pd	1–6	Spherical	UV, TEM and CV	-	(Raut et al., 2013)
Camellia sinensis	Pd	6–18	Spherical	UV, XRD, TEM and SEM	Antioxidant, antibacterial and antiproliferative ac-tivities	(Azizi et al., 2017)
Catharanthus roseus	Pd	38	Spherical	UV, XRD, FTIR and TEM	Photocatalytic activity	(Kalaiselvi et al., 2015)
Chlorella vulgaris	Pd	15	Spherical	TEM, SEM, FTIR and UV	-	(Arsiya et al., 2017)
Chrysophyllum cainito	Pd	-	Flower-like	HRTEM and XRD	Catalytic activity	(Majumdar et al., 2017)
Cinnamom zeylan-icum	Pd	15–20	Spherical	TEM, EDX, XRD and FTIR	-	(M. Sathishkumar et al., 2009)
Cinnamomum cam-phora	Pd	3.2–6	Qua-si-spherical	UV, XRD, HRTEM, EDX, SAED and XPS	-	(Yang et al., 2010)
Coffea	Pd	20–60	Spherical	TEM, UV and XRD	-	(Nadagouda & Varma, 2008)
Curcuma longa	Pd	10–15	Spherical	TEM, XRD, FTIR and EDX	-	(M Sathishkumar et al., 2009)
Delonix regia	Pd	10	-	UV, DLS, XRD, TEM, EDS and FTIR	Catalytic activity	(Dauthal & Mukhopadhyay, 2013)
Diospyros kaki	Pd	50–120	Spherical	UV, SEM and FTIR	Antibacterial activity	(Attar & Altikatoglu Yapaoz, 2018)
Euphorbia granulate	Pd	25–35	-	FTIR, UV and TEM	Catalytic activity	(Nasrollahzadeh & Mohammad Sajadi, 2016)
Filicium decipiens	Pd	2–22	Spherical	UV, TEM, XRD and FTIR	Antibacterial activity	(Sharmila et al., 2017)
Gardenia jasminoides	Pd	3–5	Spherical,	UV, XRD, FTIR and	Catalytic activity	(Jia et al., 2009)

			rod and polyhedral	TEM		
Gloriosa superba	Pd	0.8–3	Spherical	UV, HRTEM, EDS and DLS	Cytotoxic activity	(Rokade et al., 2018)
Glycin max	Pd	12	-	FTIR, XRD, HRTEM and SEM	-	(Suryawanshi et al., 2018)
Hibiscus sabdariffa	Pd	10	Spherical	UV, TEM, XRD, FTIR, SEM and EDX	Catalytic activity	(Hekmati et al., 2017)
Hippophae rham-noides	Pd	5	Spherical	XRD, SEM, TEM and UV	Catalytic activity	(Nasrollahzadeh, Sajadi, et al., 2015)
Lithodora hispidula	Pd	15.03–21.6	Rod	UV, PMS, XRD, SEM and EDX	Electrocatalytic activity	(Turunc et al., 2017)
Moringa oleifera	Pd	2–18	Spherical	UV, TEM, Zeta poten-tial, TGA, SEM and FTIR	Catalytic and biological activities	(Anand & Ranjan, 2016)
Musa paradisica	Pd	50	-	UV, SEM-EDS, XRD, DLS and FTIR	-	(Bankar et al., 2010)
Ocimum sanctum	Pd	10–80	-	PXRD, SEM, TEM, EDX, XPS, H2-TPR and SAA	Catalytic activity	(Saikia et al., 2016)
Origanum vulgare	Pd	2.2	Spherical	UV, FTIR, XRD, TEM, EDX and TGA	Catalytic activity	(Rafi Shaik et al., 2017)
Pimpinella tirupati-ensis	Pd	12.25	Spherical	UV, FTIR, XRD and TEM	Photocatalytic activity	(Narasaiah, Kumar Mandal, et al., 2017)
Pinus resinosa	Pd	3.25	Spherical	FTIR, UV, ICPAE, XPS, TEM and XRD	Catalytic activity	(G. Liu et al., 2017)
Piper nigrum	Pd	2–7	Spherical	XRD, FESEM, EDS, TEM, ATR-IR, UV, BET, TGA and ICP-OES	Aryl halide cyanation and hiyama cross-coupling reaction	(Kandathil et al., 2018)
Prunus yedoensis	Pd	50–150	Spherical	UV, XRD and FTIR	Antibacterial activity	(Manikandan et al., 2016)
Pulicaria glutinosa	Pd	20–25	-	UV, XRD, TEM, EDX and FTIR	Catalytic activity	(Khan et al., 2014)
Sapium sebiferum	Pd	2–5	Spherical	UV, FTIR, XRD, SEM, HRTEM, TGA and DLS	Photocatalytic and anti-bacterial activities	(Tahir et al., 2016)
Solanum trilobatum	Pd	60–70	Spherical	UV, FTIR and SEM	-	(Kanchana et al., 2010)
Trigonella foe-num-graecum	Pd	20–50	Spherical	UV, SEM, SAED, FTIR and XRD	Catalytic activity	(Mallikarjuna et al., 2017)

Table S13. The Plant-Mediated Synthesis of Silver and Silver Oxide Nanoparticles.

Plant Species	Nanopar-ticle	Particle Size (nm)	Morphology	Characterisation	Application	Reference
Achillea bieber-stennii	Ag	10–40	Spherical & Pentagonal	UV, FTIR, TEM and DLS	Cytotoxic activity	(Baharara et al., 2015)
Achyranthes aspera	Ag	30–80	Spherical	UV, SEM and XRD	-	(Prasad Peddi & Abdallah Sadeh, 2018)
Albizia lebbeck	Ag	12	Spherical	FTIR, UV and SEM	-	(Félix-Domínguez et al., 2019)
Allium cepa	Ag	34	Spherical	UV, DLS and TEM	Antibacterial activity	(Saxena et al., 2010)
Allium sativum	Ag	23–61	Spherical	SEM, EDX, XRD and UV	Antimicrobial activity	(Bouqellah et al., 2019)
Aloe vera	Ag	70	Triangular & Spherical	SEM, DLS, FTIR and UV	Antifungal activity	(Medda et al., 2015)
Alstonia scholaris	Ag	15–38	Spherical	UV, XRD, TEM,	-	(Shimpi et al., 2015)

				FESEM, PL and FTIR		
Althaea officinalis radix	Ag	112	Spherical	TEM, DLS and AAS	-	(Korbekandi et al., 2016)
Ananas comosus	Ag	12	Spherical	UV, EDAX, SAED, HRTEM and XRD		(Ahmad & Sharma, 2012)
Artemisia annua	Ag	-	-	UV	Antimicrobial, antioxidant and corrosion inhibition potentials	(Johnson et al., 2014)
Artemisia vulgaris	Ag	27–53	Spherical	UV, SEM, EDX, TEM, AFM and FTIR	Antibacterial, antioxidant and cytotoxic activities	(Rasheed et al., 2017)
Asclepias curassavica	Ag	75–95	Spherical	FTIR, XRD, PSA and UV	-	(Rajesh et al., 2018)
Azadirachta indica	Ag	200	Triangular	UV, DLS, SEM, TEM, EDS and FTIR	Antimicrobial and toxic activities	(Banerjee et al., 2014)
Berberis vulgaris	Ag	30–70	Spherical	UV, XRD, TEM and DLS	Antibacterial activity	(Behravan et al., 2019)
Boerhaavia diffusa	Ag	25	Spherical	SEM-EDAX, XRD, TEM, UV and FTIR	Antibacterial activity	(Vijay Kumar et al., 2014)
Brassica oleracea	Ag	30–50	Spherical	UV, XRD, SEM and EDAX	Antimicrobial activity	(R. Tamileswari et al., 2015)
Bryophyllum pinnatum	Ag	70–90	Spherical	UV, XRD, FTIR, SEM and TEM	Antibacterial activity	(Borah, 2012)
Butea monosperma	Ag	35	Spherical	FTIR, XRD, SAED, HRTEM and DLS	Cytotoxic and antibacterial activities	(Pattanayak et al., 2017)
Calliandra haematocephala	Ag	70	Spherical	SEM, EDS, XRD, UV and FTIR	Antibacterial and hydrogen peroxide sensing capability	(Raja et al., 2017)
Callistemon lanceolatus	Ag_2O	3–30	Oval & Hexagonal	UV, FTIR, XRD, SEM-EDX and HRTEM	Antioxidant and cytotoxic activity	(Ravichandran et al., 2016)
Calotropis gigantea	Ag	12–84	-	UV, SEM, XRD and FTIR	-	(Sivakumar et al., 2011)
Camellia sinensis	Ag	65–70	-	XRD, SEM, UV, DLS and Zeta potential	Antimicrobial and rheological properties	(Nakhjavani et al., 2017)
Carica papaya	Ag	-	-	UV and FTIR	Colorimetric detection of mercury ions	(Firdaus et al., 2017)
Cassia auriculata	Ag	50–70	Spherical	UV, TEM, EDS, XRD and FTIR	Antibacterial activity	(Srinivasan et al., 2015)
Cassia toral	Ag	-	Spherical	UV, FTIR, TEM and XRD	Antimicrobial activity	(Shaikh et al., 2019)
Catharanthus roseus	Ag	35–55	Spherical	UV, XRD, FTIR, EDX and SEM	Antilarvicidal activity	(Rajagopal et al., 2015)
Centella asiatic	Ag	18–21	-	UV, XRD and TEM	Antibacterial activity	(Saikia et al., 2014)
Centella asiatica	Ag_2O	11–12	Spherical	UV, FTIR, XRD, SEM and EDAX	Antimicrobial and photocatalytic activity	(Rashmi et al., 2020)
Chenopodium album	Ag	10–30	Quasi-spherical	UV, TEM, XRD, EDX and FTIR	-	(Dwivedi & Gopal, 2010)
Chrysanthemum indicum	Ag	38–72	Spherical	UV, XRD, TEM and EDX	Antibacterial and cytotoxic activities	(Arokiyaraj et al., 2014)
Chrysanthemum morifolium	Ag	20–50	Spherical	UV, XRD, TEM and FTIR	Antibacterial activity	(He et al., 2013)
Cinnamomum camphora	Ag	55–80	Triangular & Spherical	UV, XRD, SEM, TEM, AFM and	-	(Huang et al., 2007)

				FTIR		
Cinnamon zeylanicum	Ag	8–20	Spherical	UV and TEM	-	(Saliem & Ibrahem, 2018)
Citrus limon	Ag	8–15	-	FTIR, UV, TEM, SEM and AFM	Antimicrobial activity	(Vankar & Shukla, 2012)
Citrus sinensis	Ag	91	Spherical	FTIR, UV, SEM, EDS and TEM	Antibacterial activity	(Awad et al., 2014)
Clitoria ternatea	Ag	10–50	Spherical	UV, XRD, FTIR and SEM	Antibacterial activity	(Krithiga et al., 2015)
Corchorus olitorus	Ag	-	-	UV and XRD	Antibacterial activity	(Eya'Ane Meva et al., 2017)
Crocus sativus	Ag	12–20	Spherical	UV, FTIR, XRD and TEM	Antibacterial activity	(Bagherzade et al., 2017)
Cuminum cyminum	Ag	3–20	Spherical	UV, SEM, FTIR and XRD	Biological activities	(Karamian & Kamalnejad, 2019)
Cycas revoluta	Ag	2–6	Spherical	XRD, TEM, UV and SAED	-	(Jha & Prasad, 2010)
Cynara scolymus	Ag	200–223	Spherical	SEM, EDX and FTIR	Cytotoxic activity	(Erdogan et al., 2019)
Dalbergia sissoo	Ag	5–55	Spherical	UV, XRD, FTIR and TEM	-	(Singh et al., 2012)
Datura metel	Ag	25–40	-	UV, FTIR and XRD	-	(Banupriya & Muthu, 2016)
Datura stramonium	Ag	15–20	Spherical	UV, XRD, TEM, EDAX and FTIR	Antibacterial activity	(Gomathi et al., 2017)
Delonix elata	Ag	40–90	Spherical	UV and SEM	Antioxidant and toxic activities	(Reddy, 2019)
Desmodium triflorum	Ag	5–20	Spherical	UV, HRTEM and XRD	Antimicrobial activity	(Ahmad et al., 2011)
Eclipta prostrate	Ag	2–6	Spherical	UV, FTIR, XRD and TEM	-	(Jha et al., 2009)
Emblica officinalis	Ag	10–20	Spherical	UV, FTIR, TEM and SAED	-	(Ankamwar et al., 2005)
Eriobotrya japonica	Ag	20	Spherical	UV, DLS, SEM, EDX, XRD and FTIR	Antibacterial activity	(Rao & Tang, 2017)
Eucalyptus camaldulensis	Ag	16–68	Spherical	UV, FTIR, XRD, SEM, EDX and DLS	Antioxidant activity	(Alghoraibia et al., 2019)
Eucalyptus globulus	Ag	30–36	Spherical	UV, XRD, HRTEM and FTIR		(Balamurugan & Saravanan, 2017)
Eucalyptus leucoxylon	Ag	50	Spherical	XRD, SEM, TEM and UV	Antioxidant activity	(Rahimi-Nasrabadi et al., 2014)
Eucalyptus oleosa	Ag	21		SEM, EDX, DLS and FTIR		(Pourmortazavi et al., 2014)
Excoecaria agallocha	Ag	23–42	Spherical & Hexagonal	UV, XRD, FESEM and EDX	Antibacterial, antioxidant and cytotoxic activities	(Bhuvaneswari et al., 2017)
Ficus benghalensis	Ag_2O	42.7–51.4	Spherical	FTIR, UV, XRD and HRTEM	Antibacterial activity	(Manikandan et al., 2017)
Ficus carica	Ag	10–30	Spherical	UV, TEM, DLS and XRD	Antioxidant activity	(Kumar et al., 2016)
Ficus religiosa	Ag	5–50	Spherical	AFM, XRD, FTIR, FESEM, EDAX and TGA	-	(Saware & Venkataraman, 2014)
Gmelina arborea	Ag	8–32	Spherical	UV, TEM, SAED and EDX	Photocatalytic activity	(Saha et al., 2017)
Helicteres isora	Ag	8–20	Spherical	UV, TEM, DLS, XRD and FTIR	Antimicrobial activity	(Mapara et al., 2015)
Hibiscus rosa-sinensis	Ag	-	Spherical	UV and SEM	-	(Reveendran et al., 2016)

Ipomoea batatas	Ag	-	Spherical	FTIR, UV, XRD, FESEM and EDX	Antibacterial activity	(Joshi & Chhabra, 2019)
Jatropha curcas	Ag	15–50	Spherical	HRTEM, XRD and UV	-	(Bar et al., 2009)
Juglans regia	Ag	3–50	Spherical	UV, DLS, EDX, FTIR, XRD and TEM	Biological activities	(Abbasi et al., 2017)
Justica adhatoda	Ag	20–40	Spherical	UV, SEM, EDX and FTIR	-	(Bharathi et al., 2015)
Lantana camara	Ag	13	Spherical	UV, FTIR and TEM	Antibacterial activity	(Sivakumar et al., 2012)
Lawsonia inermis	Ag	50	Spherical	UV, AFM and FTIR	Solar cell	(S.C.G. Kiruba Daniel et al., 2013)
Lens culinaris	Ag	5–25	Spherical	ICP, XRD and TEM	Antibacterial activity	(Shams et al., 2014)
Lycium barbarum	Ag	3–15	Spherical	UV, TEM and XRD		(Dong et al., 2017)
Macrotyloma uniflorum	Ag	12	Spherical	XRD, TEM, UV and FTIR		(Vidhu et al., 2011)
Melia azedarach	Ag	34–48	Spherical	UV, SEM and EDX	Antibacterial activity	(Mehmood et al., 2017)
Melia dubia	Ag	5–35	Spherical	UV, XRD and SEM-EDS	Cytotoxic activity	(Kathiravan et al., 2014)
Memecylon edule	Ag	50–90	Cubic	UV, SEM, TEM, EDAX and FTIR		(Elavazhagan & Arunachalam, 2011)
Mentha piperita	Ag	20–50	Spherical, triangular, hexagonal & irregular	FTIR, UV, AFM, TEM and DLS	Antibacterial activity	(Gabriela et al., 2017)
Mimusops elengi	Ag	55–83	Spherical	UV, FTIR and SEM	Antibacterial activity	(Prakash et al., 2013)
Morinda citrifolia	Ag	100		UV	Antimicrobial activity	(Pai et al., 2015)
Musa balbisiana	Ag	200	Spherical	UV, DLS, SEM, TEM, EDS and FTIR	Antimicrobial and toxic activities	(Banerjee et al., 2014)
Musa paradisiaca	Ag	23.7	Spherical	UV, TEM, FESEM, EDX, XRD and FTIR	Antimicrobial activity	(Ibrahim, 2015)
Nelumbo nucifera	Ag	45	Spherical & Triangular	UV, SEM, TEM, XRD and FTIR	Antilarvicidal activity	(Santhoshkumar et al., 2011)
Nerium oleander	Ag	10–40		UV, FTIR and SEM		(Bharathi, 2017)
Ocimum sanctum	Ag	14.6	Spherical	PL, FTIR, XRD and TEM	Antibacterial activity	(Jain & Mehata, 2017)
Ocimum tenuiflorum	Ag	200	Cubic	UV, DLS, SEM, TEM, EDS and FTIR	Antimicrobial and toxic activities	(Banerjee et al., 2014)
Olea europaea	Ag	10–30	Cubic	UV, SEM, XRD, AAS and FTIR	Antibacterial activity	(M. Awwad et al., 2013)
Vaccinium oxycoccus	Ag	1.4–8.6	Spherical	TEM, Zeta potential, UV and FTIR	Antimicrobial activity	(Ashour et al., 2015)
Parthenium hysterophorus	Ag	5–25	-	UV, PSA and FTIR	Antibacterial and antioxidant activities	(Kalaiselvi et al., 2013)
Pelargonium hortorum	Ag	25–150	Spherical	DLS, Zeta potential, UV, FTIR and STEM	-	(Rivera-Rangel et al., 2018)
Phoenix dactylifera	Ag	14–30	Spherical	UV, SEM, HRTEM and DLS	Antibacterial activity	(Ansari & Alzohairy, 2018)
Phyllanthus niruri	Ag	15	Cubic, rectangular, triangular and spherical	UV, FTIR and SEM	Antibacterial activity	(Amalorpavamary et al., 2019)
Pinus longifolia	Ag_2O	-	Triangular &	UV and SEM	Antibacterial activity	(Lawrence, 2015)

			Spherical			
Piper betle	Ag	-	-	UV and FTIR	Antibacterial activity	(Praba et al., 2016)
Piper pedicellatum	Ag	3–30	Spherical	TEM, XRD, UV and HRTEM	Photocatalytic activity	(Tamuly, Hazarika, Bordoloi, et al., 2013)
Pistacia atlantica	Ag	50	Spherical	UV, XRD, TEM, SEM and EDX	Antibacterial activity	(Golabiazar et al., 2019)
Poaceae	Ag	15	Spherical	UV, XRD and TEM	Antibacterial, antifungal and cytotoxic activities	(Khatami et al., 2018)
Pongamia pinnata	Ag	20–50	Spherical	UV, XRD, TEM and FTIR	Antibacterial activity	(Raut et al., 2010)
Premna serratifolia	Ag	23	Cubic	SEM, FTIR and XRD	Cytotoxic activity	(Arockia John Paul et al., 2015)
Prunus armeniaca	Ag	5–40	Spherical	UV, FTIR, SEM, EDX, XRD and AA	Antibacterial activity	(Islam, Amin, et al., 2019)
Prunus japonica	Ag	24	Spherical, hexagonal & irregular	UV, HRTEM, SEM, EDS, XRD and FTIR	Antibacterial and antioxidant activities	(Saravanakumar et al., 2017)
Prunus persica	Ag	40–98	Spherical	FESEM, UV and XRD	Antimicrobial and catalytic activities	(R. Kumar et al., 2017)
Psidium guajava	Ag	-	-	EDX	Antibacterial activity	(Venugopal, 2017)
Quercus brantii	Ag	6	Spherical	TEM, DLS and AAS	-	(Korbekandi et al., 2015)
Quercus macrocarpa	Ag	40	Spherical	UV, FTIR, XRD, DLS, TEM and SEM	Cytotoxic activity	(Heydari & Rashidipour, 2015)
Rheum palmatum	Ag	121	Spherical & Hexagonal	UV, XRD, SEM, EDAX, EDS, HRTEM, Zeta potential and FTIR	Antibacterial activity	(Arokiyaraj et al., 2017)
Rosa rugosa	Ag	12	Spherical	UV, TEM, XRD, FTIR, Zeta potential and EDX	-	(S. Dubey et al., 2010)
Rubus glaucus	Ag	12–50	Spherical	UV, TEM, DLS, XRD and FTIR	Antioxidant activity	(B. Kumar et al., 2017)
Rumex hymenosepalus	Ag	2–40	Cubic & Hexagonal	UV, HRTEM and FTIR	-	(Rodríguez-León et al., 2013)
Salvia spinosa	Ag	19–125	Spherical	UV, XRD, FESEM, DLS and FTIR	Antibacterial activity	(Pirtarighat et al., 2019)
Securinega leucopyrus	Ag	11–20	Spherical & Oval	UV, FTIR, TEM and SEM	Antibacterial activity	(Rao Kudle et al., 2013)
Sesuvium portulacastrum	Ag	5–20	Spherical	XRD, FTIR and TEM	Antimicrobial activity	(Nabikhan et al., 2010)
Sida acuta	Ag	16	Spherical	UV, FTIR, XRD and TEM	Antibacterial activity and corrosion inhibition potential	(Idrees et al., 2019)
Solanum lycopersicum	Ag	-	Spherical	UV, TEM and XRD	Insecticidal activity	(Bhattacharyya et al., 2016)
Solanum nigrum	Ag	4–25	Spherical	UV, FTIR, SEM and TEM	Antidiabetic activity	(Sengottaiyan et al., 2016)
Solanum tricobatum	Ag	12.5–41.9	Spherical	UV, FTIR, SEM, TEM, EDX and XRD	Antibacterial and cytotoxic activities	(Ramar et al., 2015)
Solanum tuberosum	Ag	10	Spherical	UV, PL, DLS, CD, FTIR and TEM	Effect on human serum albumin	(M. S. Ali et al., 2017)
Sphagneticola Trilobata	Ag	22–26	Spherical	UV, FTIR, XRD and SEM	Antibacterial activity	(Vinay et al., 2018)
Syzygium aro-	Ag	5–40	Spherical	FTIR, TEM, UV	Cytotoxic activity	(Venugopal et al., 2017)

maticum				and EDX		
Syzygium cumini	Ag	93	Cubic	UV, SEM, EDX, XRD and FTIR	Antioxidant activity	(Banerjee & Narendhirakannan, 2011)
Tagetes erecta	Ag	10–90	Spherical, hexagonal & irregular	UV, FTIR, XRD, TEM and EDX	Antimicrobial activity	(Padalia et al., 2015)
Taxus baccata	Ag	15–50	Cubic	FESEM, UV, FTIR and XRD	-	(Asadi et al., 2018)
Tecoma stans	Ag	2–40	Spherical	UV, FTIR, XRD, EDX and TEM	Photocatalytic and antimicrobial activities	(Biswas & Rokhum, 2018)
Tectona grandis	Ag	10–30	Spherical	UV, XRD, FTIR, SEM/EDS, FESEM and TEM	Antimicrobial activity	(Rautela et al., 2019)
Tephrosia purpurea	Ag	20	Spherical	SAED, XRD, FTIR, SEM, TEM and PL	Antimicrobial activity	(Ajitha et al., 2014)
Terminalia arjuna	Ag	5.2	Spherical & Irregular	UV, DLS, TEM and FTIR	Antibacterial activity	(Ikram & Ahmed, 2015)
Thevetia peruviana	Ag	10–30	Spherical	UV, FTIR, SEM-EDS, XRD and HRTEM	-	(Nyoman Rupiasih et al., 2013)
Trachyspermum ammi	Ag	87	Triangular	UV and HRSEM	-	(Vijayaraghavan et al., 2012)
Tribulus terrestris	Ag	16–28	Spherical	TEM, AFM, XRD, FTIR and UV	Antimicrobial activity	(Gopinath et al., 2012)
Tridax procumbens	Ag_2O	11–12	Spherical	XRD, SEM, EDAX, FTIR and UV	Antimicrobial and photocatalytic activity	(Rashmi et al., 2020)
Vaccinium oxycoccus	Ag	1.4–8.6	Spherical	TEM, Zeta potential, UV and FTIR	Antimicrobial activity	(Ashour et al., 2015)
Vitex negundo	Ag	56	Spherical	UV, FTIR, XRD and SEM	Antibacterial activity	(Gunasekaran et al., 2013)
Vitis vinifera	Ag	19	Spherical	UV, DLS, EDX and TEM	Antibacterial activity	(Roy et al., 2013)
Zingiber officinale	Ag	10	Spherical	FTIR, HRTEM, HRSEM, SAED and EDX	Antibacterial activity	(Vijaya et al., 2017)
Ziziphora tenuior	Ag	20	Spherical	FTIR, UV, XRD, TEM, SEM and EDAX	-	(Sadeghi & Gholamhoseinpoor, 2015)

Table S14. The Plant-Mediated Synthesis of Platinum Nanoparticles.

**Plant Species**	**Nanoparticle**	**Particle Size (nm)**	**Morphology**	**Characterisation**	**Application**	**Reference**
Alchornea laxiflora	Pt	3.68–8.77	Cubic	UV, FTIR, HRTEM, EDX and XRD	Oxidative desulphurisation of model oil	(Olajire et al., 2017)
Anacardium occidentale	Pt	-	Irregular Rods	UV, FTIR and TEM	Catalytic and thermal activities	(Sheny et al., 2013)
Antigonon leptopus	Pt	5–190	Spherical	UV, FTIR, SEM-EDAX, TEM and XRD	-	(Ganaie et al., 2018)
Azadirachta indica	Pt	5–50	Spherical	UV, TEM and FTIR	-	(Thirumurugan et al., 2016)
Bacopa monnieri	Pt	5–20	Spherical	UV, FTIR, TEM and EDX	Neuro-rescue effect on experimental parkinsonism in zebrafish	(Nellore et al., 2013)
Barleria prionitis	Pt	1–2		UV, HRTEM, EDS,	Cytotoxic activity	(Rokade et al., 2017)

				DLS and FTIR		
Bidens Tripartitus	Pt	4	Irregular Rods	UV, FTIR, SEM-EDS, TEM and AFM	-	(Dobrucka, 2016)
Cacumen platycladi	Pt	3	Spherical	ICP, FTIR, TEM and XRD	-	(Zheng et al., 2013)
Camellia sinensis	Pt	2–4	Spherical	UV, TEM, FTIR, TGA and EDX	Photocatalytic activity	(Khalil et al., 2014)
Carica papaya	Pt	4.17–13.23	Spherical	UV, HRTEM, FTIR and SEM-EDX	-	(Olajire & Adesina, 2017)
Cerbera manghas	Pt	9.6–11.7	Spherical	FTIR, TEM and XRD	Antibacterial activity	(Rajathi & Nambaru, 2014)
Citrus aurantium	Pt	60	Nanowires	UV and TEM	Catalytic and antimicrobial activities	(Castro et al., 2015)
Cochlospermum gossypium	Pt	2.4	Spherical	SEM-EDAX, UV, XRD, FTIR and TEM	-	(Vinod et al., 2011)
Croton caudatus geisel	Pt	10	Spherical	UV, FTIR, XRD, SEM-EDX and TEM	Biological activities	(Kumar & Kala, 2018)
Curcuma longa	Pt	-	-	UV, SEM-EDX and FTIR	Catalytic activity	(Sahin & Gubbuk, 2019)
Doipyros kaki	Pt	2–12	Spherical	UV, TEM, EDX, XRD, FTIR and ICP	-	(Song et al., 2010)
Eichhornia crassipes	Pt	3.74	Spherical	TEM, DLS, Zeta potential and FTIR	-	(Anyik & Oluwafemi, 2017)
Fumariae herba	Pt	30	Hexagonal & Pentagonal	UV, FTIR, TEM and SEM-EDS	Catalytic activity	(Dobrucka, 2019)
Gloriosa superba	Pt	10	Spherical	UV, HRTEM, EDS and DLS	Cytotoxic activity	(Rokade et al., 2018)
Jatropa glandulifera	Pt	100	Dodecahedron, Cubic & Spherical	UV, FTIR, FESEM and TEM	Antibacterial activity	(Jeyapaul et al., 2018)
Jatropa gossypifolia	Pt	20	Dodecahedron & Spherical	UV, FTIR, FESEM and TEM	Antibacterial activity	(Jeyapaul et al., 2018)
Lantana camara	Pt	35	Spherical	UV, SEM-EDAX, Raman and XRD	-	(Mavukkandy et al., 2016)
Mentha x piperita	Pt	54.3	Spherical	UV, XRD, FTIR, TM and EDX	Cytotoxic activity	(C. Yang et al., 2017)
Ocimum sanctum	Pt	2	Irregular	UV, FTIR, XRD, SEM and TEM	-	(Prabhu & Gajendran, 2017)
Phoenix dactylifera	Pt	1.3–2.6	Spherical	FTIR, XRD, SEM-EDX, TGA, UV and TEM	Cytotoxic activity	(Al-Radadi, 2019)
Prunus x yedoensis	Pt	10–50	Oval and Spherical	UV, XRD, FTIR and TEM	Antifungal activity	(Velmurugan et al., 2016)
Punica granatum	Pt	20–100	Spherical	UV, TEM, XRD, FESEM and FTIR	Cytotoxic activity	(Şahin et al., 2018)
Quercus glauca	Pt	5–15	Spherical	UV, FTIR, TEM and XRD	Electrochemical oxidation of hydrazine in water	(Karthik et al., 2016)
Sapindus mukorossi	Pt	2–19	Spherical	TEM, XRD, SEM and EDS	-	(M. N. Kumar et al., 2017)
Taraxacum laevigatum	Pt	2–7	Spherical	UV, XRD, TEM, SEM, EDX, DLS and FTIR	Antibacterial activity	(Tahir et al., 2017)
Terminalia chebula	Pt	4	Cubic & Spherical	HRTEM, SAED, EDAX and XRD	-	(Kumar et al., 2013)

Xanthium strumarium	Pt	22	Cubic & Rectangular	UV, FTIR, XRD, SEM-EDAX and TEM	Biological activities	(Kumar et al., 2019)
Zingiber officinale	Pt	-	-	UV, SEM-EDX and FTIR	Catalytic activity	(Sahin & Gubbuk, 2019)

Table S15. The Plant-Mediated Synthesis of Gold Nanoparticles.

Plant Species	Nanoparticle	Particle Size (nm)	Morphology	Characterisation	Application	Reference
Acer pentapomicum	Au	19–24	Spherical	UV, SEM, EDX, XRD and FTIR	Antibacterial, antifungal and antioxidant activities	(Khan et al., 2018)
Achillea eriohora	Au	56	Spherical	UV, XRD and SEM	-	(Elia et al., 2014)
Aerva lantana	Au	18	Spherical	UV, FTIR, XRD and HRTEM	Catalytic activity	(Siby & Beena, 2015)
Allium cepa	Au	100	Cubic & Spherical	UV, XRD, SEM and TEM	-	(Parida et al., 2011)
Aloe vera	Au	441	Spherical	UV, XRD and PSA	-	(Muralikrishna et al., 2014)
Ampelopsis grossedentata	Au	-	Spherical	UV and TEM	-	(Qing-Quan et al., 2016)
Anacardium occidentale	Au	10–30	Spherical	UV, TEM, FTIR and XRD	Cytotoxic and antimicrobial activities	(Sunderam et al., 2019)
Annona muricata	Au	25.5	Spherical	UV, TEM and FTIR	Antimicrobial activity	(Folorunso et al., 2019)
Anthriscus sylvestris	Au	18	Spherical	UV, XRD and SEM	-	(Elia et al., 2014)
Artocarpus heterophyllus	Au	20–25	Spherical	UV, SEM and FTIR	Antimicrobial activity	(Basavegowda et al., 2015)
Aspalathus hispida	Au	34	Hexagonal, Pentagonal & Spherical	DLS, HRTEM, EDX and UV	-	(Elbagory et al., 2016)
Aspalathus linearis	Au	61	Hexagonal	DLS, HRTEM, EDX and UV	-	(Elbagory et al., 2016)
Asparagus rubicundus	Au	28	Triangular & Spherical	DLS, HRTEM, EDX and UV	-	(Elbagory et al., 2016)
Azadirachta indica	Au	25–30	Spherical	AFM, TEM, UV and FTIR	Antimicrobial activity	(Rao et al., 2017)
Berberis vulgaris	Au	5–15	Spherical	UV, TEM and XRD	-	(Abootorabi et al., 2016)
Cacumen platycladi	Au	15	Spherical	UV, XRD, TEM and TGA	-	(Zhan et al., 2011)
Camellia japonica	Au	20	Spherical	UV, FTIR, XRD and SEM	Antimicrobial activity	(T. S. K. Sharma et al., 2019)
Camellia sinensis	Au	10	-	UV and TEM	-	(Banoee et al., 2010)
Cassia auriculata	Au	15–25	Triangular & Spherical	UV, TEM, XRD, SEM-EDAX and FTIR	-	(Ganesh Kumar et al., 2011)
Chenopodium album	Au	10–30	Quasi-spherical	TEM, XRD, UV, EDX and FTIR	-	(Dwivedi & Gopal, 2010)
Chenopodium formosanum	Au	8	Spherical	HRTEM, SAED, EDS, XRD and FTIR	Antibacterial activity	(Chen et al., 2019)
Cicer arietinum	Au	25	Triangular	UV, TEM, EDS, XRD, XPS, FTIR and ESI-MS	-	(Ghule et al., 2006)
Cinnamomum camphora	Au	55–80	Spherical	UV, XRD, TEM, SEM, AFM and FTIR	-	(Huang et al., 2007)
Cinnamomum verum	Au	55	-	UV, FESEM and DLS	-	(Sharma et al., 2017)
Cinnamomum	Au	25	Spherical	FTIR, HRTEM, SAED,	-	(Smitha et al., 2009)

zeylanicum				UV, XRD and PL		
Citrus maxima	Au	8–25	Spherical	UV, XRD, TEM and FTIR	Catalytic and antibacterial activity	(Yuan et al., 2017)
Citrus sinensis	Au	20–30	Spherical	AFM, TEM, UV and FTIR	Antimicrobial activity	(Rao et al., 2017)
Coffea arabica	Au	14.9	Spherical	UV, Zeta potential, DLS, FTIR, Raman, XRD and TEM	-	(Keijok et al., 2019)
Coleus amboinicus	Au	5–55	Polygonal	UV, XRD, TEM, FTIR and SAED	-	(Narayanan & Sakthivel, 2010)
Corchorus olitorius	Au	37–50	Quasi-spherical	UV, TEM, XRD, FTIR and TGA	Cytotoxic activity	(Ismail et al., 2018)
Crocus sativus	Au	5–10	Spherical	UV, TEM and XRD	-	(Abootorabi et al., 2016)
Cymbopogon citratus	Au	20–50	Triangular, Hexagonal, Rod & Spherical	FTIR, TEM, EDX and XRD	Antimalarial activity	(Murugan et al., 2015)
Cynanchum africanum	Au	99	Hexagonal, Pentagonal & Spherical	DLS, HRTEM, EDX and UV	-	(Elbagory et al., 2016)
Dicerothamnus rhinocertis	Au	63	Hexagonal	DLS, HRTEM, EDX and UV	-	(Elbagory et al., 2016)
Diospyros kaki	Au	5–300	Spherical	ICP, EDS, SEM, TEM, AFM, XPS, PSA and FTIR	-	(Song et al., 2009)
Emblica officianalis	Au	15–25	Triangular & Decahedral	UV, FTIR and TEM	-	(Ankamwar et al., 2005)
Eriocephalus africanus	Au	102	Hexagonal, Pentagonal & Spherical	DLS, HRTEM, EDX and UV	-	(Elbagory et al., 2016)
Eucalyptus globulus	Au	13–42	Spherical	UV, TEM, EDS and FTIR	-	(Dzimitrowicz et al., 2019)
Eucommia ulmoides	Au	18	Spherical	UV, HRTEM, EDX, XRD, DLS and Zeta potential	Catalytic activity	(Guo et al., 2015)
Euphorbia hirta	Au	6–71	Spherical	TEM, XRD, EDAX, AFM, PSA, FTIR and Raman	Antimicrobial activity	(Annamalai et al., 2013)
Ferula gummosa	Au	30	Spherical	UV, XRD and SEM	-	(Elia et al., 2014)
Garcinia combogia	Au	40–50	-	UV, XRD, FTIR and SEM	Antibacterial and antibiofilm activity	(Nithya, 2016)
Garcinia mangostana	Au	33	Spherical	UV, XRD and TEM	-	(Xin Lee et al., 2016)
Gnidia glauca	Au	10	Spherical	HRTEM, DLS, XRD and FTIR	Chemocatalytic activity	(Ghosh et al., 2012)
Gymnocladus assamicus	Au	5–23	Hexagonal, Pentagonal & Triangular	UV, XRD and TEM	Antibacterial activity	(Tamuly, Hazarika, Debnath, et al., 2013)
Hermannia alnifolia	Au	66	Hexagonal, Pentagonal & Spherical	DLS, HRTEM, EDX and UV	-	(Elbagory et al., 2016)
Hibiscus rosa sinensis	Au	14	Triangular, Hexagonal, Dodecahedral & Spherical	UV, TEM, XRD and FTIR	-	(Philip, 2010a)
Hibiscus sabdariffa	Au	7	Spherical	UV, XRD, TEM, FESEM, EDX, Zeta potential and FTIR	Electrooxidation of nitrite	(Mohd Taib et al., 2019)
Hypericum hookerianum	Au	34–61	-	UV, SEM-EDX and FTIR	Antiparkinson activity	(Subakanmani et al., 2015)
Indigofera brachystachya	Au	100	Hexagonal	DLS, HRTEM, EDX and UV	-	(Elbagory et al., 2016)
Lantana camara	Au	11–32	Spherical	XRD, FTIR, HRTEM and EDX	Antioxidant and cytotoxic activities	(Ramkumar et al., 2017)

Lawsonia inermis	Au	5–10	Spherical	UV, TEM, SEM-EDS, DLS and FTIR	Catalytic activity	(Abd El-Aziz et al., 2018)
Lippia citriodora	Au	2.6–50	Pentagonal & Hexagonal	DLS, NTA, SEM, EDS, TEM, ICP-OES and Zeta potential	-	(Elia et al., 2014)
Lobostemon glaber	Au	136	Triangular & Hexagonal	DLS, HRTEM, EDX and UV	-	(Elbagory et al., 2016)
Macadamia integrifolia	Au	50	Hexagonal, Triangular & Spherical	UV, XRD, SEM and EDS	Antimicrobial activity	(Dang et al., 2019)
Macrotyloma uniflorum	Au	14–17	Spherical	UV, TEM, XRD and FTIR	-	(Aromal et al., 2012)
Magnolia kobus	Au	100–300	Spherical	SEM and UV	-	(Li et al., 2012)
Mangifera indica	Au	17	Spherical	UV, TEM and XRD	-	(Philip, 2010b)
Medicago sativa	Au	30–60	Triangular, Decahedral & Icosahedral	SEM, TEM, AFM and SAED	-	(Montes et al., 2011)
Melissa officinalis	Au	20	Triangular & Spherical	UV, DLS, FTIR, SEM and TEM	-	(Dzimitrowicz et al., 2019)
Mentha piperita	Au	34	Spherical	GC-MS, UV, FESEM, DLS and FTIR	-	(Jafarizad et al., 2015)
Metalasia muricata	Au	61	Hexagonal, Pentagonal & Spherical	DLS, HRTEM, EDX and UV	-	(Elbagory et al., 2016)
Mimosa tenuiflora	Au	20–200	Hexagonal & Spherical	Zeta potential, FTIR, XPS, TEM, UV and XRD	Assessment of cytotoxicity, cellular uptake and catalysis	(Rodríguez-León et al., 2019)
Mimusops elengi	Au	9–14	Spherical	HRTEM, UV, EDX and XRD	Catalytic activity	(Majumdar et al., 2016)
Momordica cochinchinensis	Au	10–80	Triangular, Oval & Spherical	UV, FTIR, XRD, TEM and EDX	Anticoagulative and photocatalytic activities	(B. Paul et al., 2016)
Morinda citrifolia	Au	12–33	Spherical	UV, XRD, FTIR, FESEM, EDX and TEM	-	(Suman et al., 2014)
Mucuna pruriens	Au	6–18	Spherical	UV, FTIR, TEM and XRD	-	(Arulkumar & Sabesan, 2010)
Murraya koenigi	Au	20–40	Spherical	UV, FTIR, TEM, TGA and XRD	-	(Alam et al., 2014)
Nauclea latifolia	Au	7	Spherical	UV, EDX, DLS and TEM	-	(Dozie-Nwachukwu et al., 2015)
Nepenthes khasiana	Au	50–80	Spherical	UV, SEM, XRD, FTIR and TEM	Antimicrobial activity	(Bhau et al., 2015)
Nerium oleander	Au	20–40	Spherical	UV, PRS, HRTEM, XRD and DLS	Cytotoxic and catalytic activity	(Barai et al., 2018)
Nidorella foetida	Au	97	Hexagonal, Pentagonal & Spherical	DLS, HRTEM, EDX and UV	-	(Elbagory et al., 2016)
Nigella arvensis	Au	3–37	Spherical	UV, XRD, FTIR and TEM	Antibacterial, antioxidant, cytotoxic and catalytic activities	(Chahardoli et al., 2018)
Nyctanthes arbortristis	Au	20	Spherical	UV, TEM, XRD, FTIR and NMR	-	(Das et al., 2011)
Ocimum sanctum	Au	1–100	Spherical	TEM, UV and XRD	-	(Lee et al., 2016)
Ocimum tenuiflorum	Au	25–30	Spherical	AFM, TEM, UV and FTIR	Antimicrobial activity	(Rao et al., 2017)
Otholobium bracteolatum	Au	53	Hexagonal, Pentagonal & Spherical	DLS, HRTEM, EDX and UV	-	(Elbagory et al., 2016)
Papaver somniferum	Au	77	Spherical	UV, SEM and FTIR	-	(Muhammad et al., 2017)
Pelargonium cit-	Au	33.8	Polygonal, Triangu-	GC-MS, UV, FESEM,	-	(Jafarizad et al., 2015)

ronellum			lar & Spherical	DLS and FTIR		
Pelargonium graveolens	Au	6–78	-	DLS, NTA, SEM, EDS, TEM, ICP-OES and Zeta potential	-	(Elia et al., 2014)
Pelargonium zonale	Au	8–20	Spherical	UV, TEM, EDS and FTIR	-	(Franco-Romano et al., 2014)
Periploca Aphylla	Au	25–30	Spherical	UV, TEM, XRD and FTIR	-	(Kaykhaii et al., 2018)
Phyllanthus amarus	Au	65–99	Cubic & Spherical	UV, SEM, XRD, EDX, AFM, PSA and FTIR	-	(Annamalai et al., 2011)
Piper longum	Au	56	Spherical	UV, TEM-EDX, DLS and TGA	Antioxidant and catalytic activities	(Nakkala et al., 2016)
Piper nigrum	Au	55	Rectangular	UV, FESEM and DLS	-	(Sharma et al., 2017)
Pistacia integerrima	Au	20–200	-	UV, FTIR and SEM	Biological activities	(Islam, Jalil, et al., 2019)
Podocarpus falcatus	Au	102	Pentagonal	DLS, HRTEM, EDX and UV	-	(Elbagory et al., 2016)
Podocarpus latifolius	Au	54	Pentagonal & Hexagonal	DLS, HRTEM, EDX and UV	-	(Elbagory et al., 2016)
Pogestemon benghalensis	Au	10–50	Triangular & Spherical	UV, XRD, TEM and FTIR	Photocatalytic activity	(Paul et al., 2015)
Polyscias scutellaria	Au	5–20	Cubic	UV, PSA, FTIR, TEM-SAED and XRD	Catalytic activity	(Yulizar et al., 2017)
Psidium guajava	Au	15	Spherical	UV, XRD, TEM and FTIR	-	(Taha & Shamsuddin, 2013)
Punica granatum	Au	34–312	Irregular	DLS, NTA, SEM, EDS, TEM, ICP-OES and Zeta potential	-	(Elia et al., 2014)
Pyrus pyrifolia	Au	10–40	Spherical	UV, TEM, EDS, XRD and FTIR	-	(Ghodake & Lee, 2011)
Rhus coriaria	Au	21	Spherical	UV, FTIR, TEM and Zeta potential	Antioxidant activity	(Shabestarian et al., 2017)
Rosa hybrida	Au	10	Spherical, Triangular & Hexagonal	UV, FTIR, XRD, EDX, DLS and TEM	-	(Noruzi et al., 2011)
Rosa rugosa	Au	11	Spherical	UV, TEM, XRD, FTIR, Zeta potential and EDX	-	(S. Dubey et al., 2010)
Rosmarinus officinalis	Au	9–61	Spherical	UV, TEM, EDS and FTIR	-	(Dzimitrowicz et al., 2019)
Salvia africana-lutea	Au	69	Hexagonal, Pentagonal & Spherical	DLS, HRTEM, EDX and UV	-	(Elbagory et al., 2016)
Salvia officinalis	Au	4–72	Triangular	DLS, NTA, SEM, EDS, TEM, ICP-OES and Zeta potential	-	(Elia et al., 2014)
Saraca indica	Au	5–23	Polygonal	SPR, HRTEM, AFM, XRD and FTIR	Catalytic activity	(Dash et al., 2014)
Searsia dissecta	Au	68	Triangular & Spherical	DLS, HRTEM, EDX and UV	-	(Elbagory et al., 2016)
Senecio pubigerus	Au	49	Hexagonal, Pentagonal & Spherical	DLS, HRTEM, EDX and UV	-	(Elbagory et al., 2016)
Sesbania drummondii	Au	6–20	Spherical	ICP, TEM, XANES and EXAFS	Catalytic activity	(Sharma et al., 2007)
Solanum nigrum	Au	50	Spherical	UV, DLS, Zeta potential, TEM, XRD and FTIR	Antioxidant and antibacterial activity	(Muthuvel et al., 2014)
Sorbus aucuparia	Au	18	Triangular, Hexagonal & Spherical	TEM, UV, XRD, EDX and FTIR	-	(S. P. Dubey et al., 2010)
Stachys lavandulifolia	Au	22–30	Triangular & Spherical	UV, FTIR, XRD, EDS, SEM and AFM	Catalytic activity	(Veisi et al., 2018)

Stevia rebaudiana	Au	5–20	Spherical	UV, FTIR, XRD, SEM and TEM	-	(Sadeghi et al., 2015)
Syzygium aromaticum	Au	55	Cubic	UV, FESEM and DLS	-	(Sharma et al., 2017)
Tabebula argentiea	Au	56	Spherical	SEM-EDX	Cytotoxic activity	(Vinay et al., 2017)
Terminalia catappa	Au	10–35	Spherical	UV, XRD, FTIR and TEM	-	(Ankamwar, 2010)
Torreya nucifera	Au	8–42	Spherical	UV, TEM, XRD and FTIR	Cytotoxic activity	(Kalpana et al., 2013)
Wedelia trilobata	Au	10–50	Spherical	UV, XRD, FTIR, TEM-EDX and SAED	Cytotoxic activity	(Dey et al., 2018)
Zea mays	Au	30	Spherical	UV, FTIR, SEM, TEM and EDAX	-	(Jon et al., 2019)
Zingiber officinale	Au	5–15	-	DLS, UV, FTIR and TEM	Blood compatibility	(Kumar et al., 2011)
Ziziphus zizyphus	Au	40–50	Triangular & Hexagonal	TEM, SEM, AFM, XRD, UV, EDX and TGA	Antimicrobial activity	(Aljabali et al., 2018)
